# PROMIS® Health Organization (PHO) 2022 Conference Abstracts: Prague, Czech Republic, 23–24 October 2022

**DOI:** 10.1186/s41687-023-00547-1

**Published:** 2023-03-21

## O9

### Reliability and validity of the PROMIS-25 among children living with burn injuries

#### Dagmar Amtmann^1^, Kara McMullen^1^, Alyssa Bamer^1^, Andrew Humbert^1^, Colleen Ryan^2^, Jeffrey Schneider^3^, Lewis Kazis^4^, Barclay Stewart^1^, Oscar Suman^5^

##### ^1^University of Washington, Seattle, USA. ^2^Harvard Medical School, Boston, USA. ^3^Spaulding Rehabilitation Hospital, Boston, USA. ^4^Boston University, Boston, USA. ^5^University of Texas Medical Branch, Galveston, USA

*Journal of Patient-Reported Outcomes* 2023, **7(1)**: O9

**Objective**: The Patient-Reported Outcomes Measurement System (PROMIS)-25 profile has been validated for use in diverse populations of children with many conditions, though not among burn-injured children. The purpose of this study was to examine the reliability and validity of PROMIS-25 scores in children living with burn injury.

**Methods**: Data were collected through a multi-center longitudinal study of outcomes after burn injury. Each domain of the PROMIS-25 Profile was evaluated for reliability and validity. Floor and ceiling effects, unidimensionality, internal consistency using Cronbach’s Alpha, and reliability with item response theory (IRT) information functions were examined. Correlations with other measures [Post-Traumatic Growth Inventory-Child (PTGI-C), Child PTSD Symptom Scale (CPSS) and Burn Outcome Questionnaire Body Image Scale (BOQBI)] were calculated to assess concurrent validity.

**Results**: 256 children who sustained a moderate to severe burn provided responses on PROMIS-25 domains 6 months to 10 years after injury (mean 4.3, SD 4.1 years after injury). Participants’ ages ranged from 8 to18 years at time of assessment. All PROMIS-25 domains showed high internal consistency (α = 0.90–0.95). A majority of the sample reported no symptoms (anxiety [58.2%], depression [54.6%], fatigue [50.8%], pain [60.1%]). There was a large ceiling effect on peer relationships (46.8%) and physical function mobility (57.5%). One-factor confirmatory factor analyses supported unidimensionality for all domains (all CFI > 0.98). Reliability based on PROMIS IRT information functions was adequate for group mean comparisons (> 0.8) across at least some trait levels for all domains except fatigue and anxiety, which had lower reliability (< 0.8) across the entire trait range. The magnitude and direction of correlations were as hypothesized (0.32 for peer relationships and body image; 0.51 for depressive symptoms and PTSD) except for weaker than hypothesized negative correlations between PTGI-C and the anxiety and depression domains.

**Conclusions**: The results provide evidence of reliability and validity of PROMIS-25 scores among children living with burn injury. However, reliability of all domains was low to moderate. Reliability could likely be improved, and ceiling effects reduced by administering computer adaptive tests or longer short forms such as those in PROMIS-37, which includes six items per domain rather than four.

## O10

### Scarce evidence for the content validity of existing health-related quality-of-life questionnaires for children with cancer

#### Maria Rothmund^1,2^, Andreas Meryk^1^, Gerhard Rumpold^1^, Roman Crazzolara^1^, Samantha Sodergren^3^, Anne-Sophie Darlington^3^, David Riedl^1^

##### ^1^Medical University Innsbruck, Innsbruck, Austria. ^2^University of Innsbruck, Innsbruck, Austria. ^3^University of Southampton, Southampton, UK

*Journal of Patient-Reported Outcomes* 2023, **7(1)**: O10

**Objective**: Content validity, the extent to which an instrument measures what it purports to measure, is arguably the most fundamental psychometric property of measures. The COSMIN methodology defines it by relevance, comprehensiveness, and comprehensibility. To ensure content validity of patient-reported outcome measures (PROMs), development guidelines highlight the importance of patient involvement. This systematic review evaluates the development of available PROMs for health-related quality of life (HRQOL) in children with cancer and grades the evidence for their content validity.

**Methods**: In December 2020, PubMed was searched systematically to identify PROMs, which are used to assess HRQOL of children with cancer (lower age-limit > 7 and ≤ 12; upper age-limit < 21). The COSMIN methodology for assessing the content validity of PROMs was applied. It gives standardized instructions to grade the evidence for the relevance, comprehensiveness, and comprehensibility of PROMs based on quality ratings of development and content validity studies.

**Results**: Twelve PROMs were included. Most development studies were of doubtful or inadequate quality. Content validity studies were hardly available and mostly of inadequate quality. According to the COSMIN methodology, the evidence for the content validity was low or very low for almost all PROMs. Only the PROMIS Pediatric Profile had moderate evidence. In general, results indicated that the PROMs cover relevant issues, while evidence for comprehensiveness and comprehensibility was partly inconsistent or negative.

**Conclusions**: This review showed that there is scarce evidence for the content validity of almost all available PROMs for HRQOL assessment in children with cancer. The only instrument with moderate evidence for its content validity is the PROMIS Pediatric Profile. The overall lack of evidence is due to doubtful or inadequate studies with missing patient involvement and poor reporting. Further research should adhere to existing guidelines on qualitative methods and to reporting standards for development, cognitive interview, and content validity studies. This should inform the development of new instruments but also content validity studies to strengthen the evidence for existing PROMs. The methods used for the PROMIS Pediatric Profile could serve as an example or starting point for upcoming research projects.

## P13

### Functional follow-up in potentially severely injured patients: feasibility, outcome, and experience

#### Ingri Grimnes Olsen^1,2^, Mona Stedenfeldt^2^, Astrid Woodhouse^2,3^, Torunn Hatlen Nøst^2,4^, Oddvar Uleberg^5,6^

##### ^1^Department of Anesthesia and Critical Care, St. Olavs University Hospital, Trondheim, Norway. ^2^National advisory unit for complex symptom disorders, St. Olavs University Hospital, Trondheim, Norway. ^3^Department of Circulation and Medical Imaging, Norwegian University of Science and Technology, Trondheim, Norway. ^4^Department of Mental Health, Norwegian University of Science and Technology, Trondheim, Norway. ^5^Department of Emergency Medicine and Pre-hospital services, St. Olavs University Hospital, Trondheim, Norway. ^6^Department of Research and Development, Division of Emergencies and Critical Care, Oslo University Hospital, Oslo, Norway

*Journal of Patient-Reported Outcomes* 2023, **7(1)**: P13

**Objective**: To provide new knowledge regarding the potential for an electronic easy-to-use solution for long-term follow-up of potentially severely injured patients. The main objectives are to implement and test a self-reported solution using PROMIS-29, evaluate feasibility, and identify patients’ experiences on and needs for follow-up.

**Methods**: St. Olav’s University Hospital serves as a regional trauma centre for Central Norway. In this study, trauma patients are defined as patients received by a multidisciplinary trauma team (TT). All patients aged ≥ 18 years, admitted to the regional trauma centre between 13.09.2021–12.09.2022, and able to give consent and fill in the electronic questionnaires will be included. Consenting respondents are asked the questions incorporated in the PROMIS-29 and EQ-5D-5L to provide baseline values prior to trauma. Patients discharged before contact with the project coordinator will be contacted by phone. 14 days later the patients will receive a text message with an electronic link to the PROMIS-29 and EQ-5D-5L surveys. This is repeated at 3-, 6- and 12-months post-injury. After 12 months, a representative sample of consenting respondents will undergo a semi-structured interview related to their experiences related to follow-up by the health services.

**Results**: Analyses will address the following research questions: (1) Is implementation of PROMIS-29 as an electronic patient reporting outcome measure (PROM) feasible among potentially severely injured patients? (2) Is the use of PROMIS-29 capable of identifying specific domains of challenge pertaining to long-term outcome among potentially severely injured patients? and (3) What are the experiences and needs for follow-up among patients with potentially severe injuries?

**Conclusions**: This study is one of the first to introduce PROMIS-29 as an assessment tool for potentially severely injured patients. Long-term follow-up of this patient population is important and patient-reported outcome measures combined with an easy-to-use reporting solution may be vital in reducing morbidity and reduced functional ability.

## O14

### Developing IRT-based item banks measuring communicative participation: inclusion of people with communication difficulties in research

#### Eline Alons^1^, Nicole ter Wal^1^, Caroline Terwee^2^, Margareet Luinge^3^, Ellen Gerrits^1,4^, Lizet van Ewijk^1^

##### ^1^Research group Speech and Language Therapy: Participation through Communication—HU University of Applied Sciences, Utrecht, Netherlands. ^2^Department of Epidemiology and Data Science, Amsterdam University Medical Center, Amsterdam, Netherlands. ^3^Research group Healthy Aging, Allied Health Care and Nursing, Hanze University Groningen, University of Applied Sciences, Groningen, Netherlands. ^4^Clinical Language, Speech and Hearing Sciences, Utrecht University, Utrecht, Netherlands

*Journal of Patient-Reported Outcomes* 2023, **7(1)**: O14

**Objective**: To identify relevant self-experienced aspects of communicative participation in children, adolescents, and adults with speech, language, hearing, and voice disorders, to inform the development of an IRT-based item bank using the PROMIS methodology.

**Methods**: This project builds on work by Baylor and colleagues to further develop an IRT-based item bank measuring communicative participation for children, adolescents, and adults with communication disorders. In this project we explicitly target and facilitate the inclusion of adults and children with communication disorders. As the target population has language difficulties, we used a range of creative research methods for the concept elicitation study.

We used a qualitative research design, using multiple diary approaches, such as the photovoice method or diaries. These sensitizing approaches were all followed by semi-structured interviews, with supported conversation techniques, to further explore the aspects of communicative participation. All qualitative methods were adjusted to be accessible for participants with communication difficulties.

By presenting this creative and accessible method, we would like to inspire the audience to include people who have difficulties understanding, processing, and using (verbal) health information in PROM research. PROMs are heavily reliant on (verbal) language, and items are often complex in linguistic formulation. People with communication disorders, low literacy skills or other communication vulnerabilities not only struggle using PROMs, but they are almost always excluded from PROM research, as adequate language ability is often an inclusion criterium.

**Results**: The concept elicitation in adults revealed over 40 concepts, divided in 6 themes. Many of these concepts had not previously been identified in a systematic literature search. The data collection for children and adolescent is ongoing and will also be reported at the conference.

**Conclusions**: This study highlights the importance of accessibility of PROM research from the onset of instrument development. It also provides an example on how researchers can include participants that are communicatively vulnerable, a group that is now categorically excluded from PROM development.

## O15

### Establishing governance to improve the use of PROMIS in the clinical setting

#### Mark Nyman, Stacy Schmitt, Martha Springsted, Sasha Ford, Jenelle Meyer, Lori Steinke, Rachel Martin

##### Mayo Clinic, Rochester, USA

*Journal of Patient-Reported Outcomes* 2023, **7(1)**: O15

**Objective**: Improving the impact of patient reported outcomes (PROs) in the clinical setting is a necessary evolution of PRO development and use. The objective is to describe the collaboration between business service resources and multiple stakeholder specialties utilizing PROs that culminated in creation of a governance model that standardized collection methods, visualizations, and reporting, while transitioning from disease-specific legacy PROs to PROMIS CAT.

**Methods**: Collaborative efforts between practice specialties, Health Information Management Services, IT/Epic Reporting, IT/Epic Build, Registry Services, and Management Engineering & Consulting identified a governance model to support current and future PRO use at Mayo Clinic.

**Results**: The Mayo Clinic ePRO Collaborative was established with the following objectives: (1) Identify users and current state of PRO collection; (2) Collect priorities and determine commonalities to further PROs through shared knowledge and resources; (3) Serve as a knowledge base for future PRO stakeholders; (4) Develop and govern PRO standards; (5) Partner with external vendors to improve PRO functionality and collection; (6) Prioritize and develop analytics for quality assurance and improvement; and (7) Enhance care delivery through proof-of-concept care coordination teams responding to completed PROs. The ePRO Collaborative inventoried user PRO collection, system functionality, challenges and barriers, priorities, and resources. Common and redundant themes were recognized and a workplan was created to improve PRO use in the clinical setting. Processes for future PRO requests were identified whereas the ePRO Collaborative would serve as a consulting team in the technical requirements phase of PRO approval and encourage use of PROMIS as a universally applicable PROM over legacy disease-specific PROMs. The Mayo Clinic ePRO collaborative has been successful in leveraging best practice in ePRO implementation across the enterprise. Several enterprise departments have implemented use of several PROMIS-CAT cross-cutting domains. Use of common domains has reduced patient facing redundancy. Standardization has assisted with simplification of build and more efficiently used available resources. The collaborative has been an excellent source of consensus building for build enhancements.

**Conclusions**: Following Epic go-live in 2018, specialties at Mayo Clinic were eager to implement PROMs through programmatic functionality within the Epic EHR. Each individual practice area acquired resources independently to build, implement, and monitor PROMs creating redundancy across the enterprise and inconsistent clinical use. By establishing the ePRO Collaborative, practice specialties and shared business service resources partner to standardize use and prioritize optimizations to improve patient acceptance and clinical use which are contributing to fulfilling the quality value equation.

## O18

### PROPr vs EQ-5D and QLU-C10D—comparisons in clinical and general population samples

#### Christoph Paul Klapproth, Matthias Rose, Felix Fischer

##### Charité University Medicine, Berlin, Germany

*Journal of Patient-Reported Outcomes* 2023, **7(1)**: O18

**Objective**: The PROMIS Preference Score (PROPr) is a health state utility (HSU) score using PROMIS as underlying descriptive system for the calculation of quality-adjusted life years (QALY) in cost-effectiveness analyses. It claims to measure HSU less coarsely and more comprehensively than existing measures such as the EQ-5D. We compared PROPr to other HSU scores in respect of convergent validity and known-group validity as well as ceiling/floor effects and agreement in different clinical and general population samples.

**Methods**: We measured HSU using the PROPr, the EQ-5D-5L or -3L, and/or the EORTC QLU-C10D in patient samples of rheumatology and psychosomatic medicine (n = 141), breast cancer (n = 291), other cancers (n = 420) and low back pain (n = 218) as well as general population samples in Germany, France, and the United Kingdom (each n = 1,500). We investigated agreement using Pearson (r) and intra-class correlation coefficients (ICC) as well as Bland–Altman plots and the upper/lower quintile for the measurement of ceiling/floor effects as measures of comparison.

**Results**: The mean PROPr was substantially lower than the mean EQ-5D-5L, -3L or QLU-C10D (d = 0.18–0.35). This difference between the PROPr and the other HSU scores was generally invariant to sex, age, education, occupation, treatment and condition. The PROPr did not show ceiling effects but considerable floor effects in patient samples (up to 42%) and an approximate normal distribution in general population samples. The EQ-5D-5L, -3L, and QLU-C10D showed large ceiling effects in all samples (up to 86%). Correlation between PROPr and QLU-C10D (r = 0.80–0.83) was higher than between PROPr and EQ-5D-5L or -3L (r = 0.66–0.74). Agreement in terms of ICC was low to moderate (0.27–0.48). The PROPr was more (less) efficient than the EQ-5D-3L in detecting differences between clinically less (more) severe groups of patients with low back pain.

**Conclusions**: The PROPr measures HSU substantially different from the EQ-5D-5L, -3L, and the QLU-C10D, but this difference is constant across subgroups. This is a result of the PROPr’s multiplicative multi-attribute utility function and the broad underlying PROMIS scales. QALY calculated with different scores can therefore not be used interchangeably. Future research should examine the PROPr’s longitudinal performance, including responsiveness to change and test–retest reliability.

## P19

### The association between PROMIS functional outcomes and quality-of-life in patients with proximal humeral fractures

#### Tim Kobes^1,2^, Sandra Wilson^1^, Ruben Hoepelman^1,2^, Marilyn Heng^1^

##### ^1^Massachusetts General Hospital, Boston, USA. ^2^University Medical Center Utrecht, Utrecht, Netherlands

*Journal of Patient-Reported Outcomes* 2023, **7(1)**: P19

**Objective**: (1) To investigate the correlation between physical function and quality of life; and (2) to assess the effect of surgical treatment on functional outcomes in elderly and non-elderly patients with proximal humerus fracture.

**Methods**: We performed a prospective cohort study. All patients (≥ 18 years) with a proximal humerus fracture were eligible for inclusion. Patients were divided by age: elderly (≥ 65 years) and non-elderly (< 65 years). Patients completed the PROMIS physical function, PROMIS upper extremity, PROMIS global health, EQ-5D, and the Disabilities of the Arm, Shoulder and Hand (DASH) questionnaires at multiple time points after injury. Follow-up was up to 12 months. Correlation was assessed using the Pearson correlation coefficient or Spearman rho. Linear regression was adjusted for functional outcomes and Neer classification.

**Results**: Between 2018 and 2021, 83 patients were included (86% female). Thirty-six patients (49%) were 65 years or older, one- and two-part fractures accounted for 67% of fractures, and 68 patients (82%) were treated conservatively. The EQ-5D was significantly correlated with PROMIS physical function, PROMIS upper extremity, and the DASH score at all time points in both elderly and non-elderly patients. Correlations were stronger in elderly patients except at the 12-month time-point. Surgical treatment was significantly associated with lower PROMIS Mental scores in elderly patients 12 months after injury (β-coefficient − 10.1 to − 11.9; *p* < 0.05) after controlling for Neer classification. In non-elderly patients, there was no similar association.

**Conclusions**: In elderly patients with a proximal humerus fracture, quality of life is correlated with physical function, mainly in the first 6 months after injury. In surgically treated elderly patients, mental health scores were significantly lower than in conservatively treated elderly. These findings suggest that a holistic approach of elderly patients with a proximal humerus fracture is warranted to consider quality of life, physical function, and mental health outcomes.

## O20

### Challenges and successes in the implementation of PROMs at a musculoskeletal specialty hospital

#### Vinicius C. Antao^1^, Sandhya Ghanta^2^, Brian Chicosky^2^, Chandra Gantha^2^, Catherine MacLean^1^

##### ^1^Hospital for Special Surgery, Center for the Advancement of Value in Musculoskeletal Care, New York, USA. ^2^Hospital for Special Surgery, Department of Information Technology, New York, USA

*Journal of Patient-Reported Outcomes* 2023, **7(1)**: O20

**Objective**: To develop a framework to collect PROMs as standard-of-care at a musculoskeletal specialty hospital.

**Methods**: We compiled an inventory of PROMs collected through registries and other platforms and obtained stakeholder consensus on a short list of PROMs for relevant domains and conditions.

We custom built each PROM into the electronic health record (EHR) (Epic) as flowsheets, including automatic score calculation. We used PROMIS instruments as provided by Epic’s App Orchard but built flowsheet rows to store scores. We assigned PROMs through several methods: “silent” Best Practice Advisory and Procedure Pass (triggered by surgery/visit scheduling or specific procedures/conditions); bundled with other questionnaires (cascading after conditional logic); or assigned through order sets (when logic was too complex/subjective—e.g., verbal vs. non-verbal pediatric patients).

We made PROMs scores available at the point-of-care through Synopsis or print groups, created specific “dot-phrases” to allow for importing scores into clinical notes, and mapped all PROMs data to be seamlessly transferred to Epic’s data warehouse, where they can be combined with other clinical data for operational (e.g., completion dashboards) and research purposes.

We created provider tip sheets and a patient video to encourage use and completion of PROMs.

**Results**: Since 2017, we implemented 55 unique PROMs, including 11 PROMIS Banks, one Short Form, and one Profile, for 13 service lines. While we initially employed a call center to collect the PROMIS Global Health, it proved resource intensive and not scalable for condition-specific PROMs. Therefore, we changed the reminder email language, provided a direct link to questionnaires and the video (which had ~ 50,000 views), observing a significant increase in monthly completion rates through the portal (60–82%) compared to the period before these changes (32–45%). Because Epic does not currently allow PROMIS CATs to be bundled with other questionnaires, we built a decision tree at registration level, to allow for condition-specific assignments.

**Conclusions**: In our journey to implement PROMs as a standard-of-care, we used existing EHR resources and creativity to overcome operational obstacles. The benefits of collecting PROMs electronically through EHRs outweigh the complexities of implementation.

## P21

### Classifying individuals with chronic low back pain using the Impact Stratification Score

#### Anthony Rodriguez^1^, Patricia Herman^2^, Maria Edelen^1,3^, Ron Hays^4^

##### ^1^RAND Corporation, Boston, USA. ^2^RAND Corporation, Santa Monica, USA. ^3^Patient Reported Outcomes, Value and Experience (PROVE) Center, Department of Surgery, Brigham and Women’s Hospital, Boston, USA. ^4^Division of General Internal Medicine & Health Services Research, UCLA Department of Medicine, Los Angeles, USA

*Journal of Patient-Reported Outcomes* 2023, **7(1)**: P21

**Objective**: Develop and evaluate a pain impact classification scheme derived for the Impact Stratification Score (ISS) and test against the current classification by examining cross-sectional and longitudinal data to identify the version that is best at grouping individuals based on current severity and prognosis.

**Methods**: The sample of 1965 respondents who indicated having chronic lower back pain, had an average age of 41.1, 51.8% male, and 85% White. Study participants completed the PROMIS-29 v2.1 profile survey that contains the 9 ISS items (4 Physical Function, 4 Pain Interference, and Pain intensity). Respondents also completed the Roland-Morris Disability Questionnaire and items asking about overall health, whether pain has limited life and/or work, and whether poor health has resulted in unemployment. Latent profile analysis (LPA) was used to identify an optimal solution using the nine ISS items. Information criteria (-2LL, AIC, BIC, aBIC) and likelihood ratio tests (Lo-Mendell-Rubin and Vuong-Lo-Mendell-Rubin) were used to decide on an optimal solution and inform the classification scheme. We then compared associations with current severity and prognosis between the originally proposed ISS classification and the new classification.

**Results**: LPA identified four pain impact groups: no to mild with low pain intensity, no to mild with higher pain intensity, moderate, and severe. Emergent classes roughly aligning with mean scores across the physical function and interference items with pain intensity mainly differentiating at the lowest levels of pain impact. Scores were then computed, and respondents assigned to corresponding pain impact groups. Cross-sectional and longitudinal analyses indicate that the new 4-group classification shows promise for greater discrimination among individuals at the extremes.

**Conclusions**: This study presents evidence for an alternate, empirically based, classification scheme which demonstrates potential for differentiating severity and prognosis.

## O25

### Can PROMIS scores be used to eliminate unnecessary follow up clinic visits after surgery?

#### Jefferson Hunter, Gabriel Ramirez, Caroline Thirukumaran, Judith Baumhauer

##### University of Rochester School of Medicine and Dentistry, Rochester, USA

*Journal of Patient-Reported Outcomes* 2023, **7(1)**: O25

**Objective**: To use PROMIS scores to assess a trend in pain improvement after surgery suggestive of a successful outcome and eliminate additional follow up visits; to calculate cost savings using this methodology based on prior practice patterns.

**Methods**: Retrospective PROMIS Pain Interference (PI) data were obtained for common elective foot (n = 832) and ankle (n = 851) surgeries. Patients were categorized into quartiles based on pre-operative PI score with Quartile 1 (Q1) representing 25% of patients with the lowest PI scores. Minimal clinically important difference (MCID) was defined by as ½ standard-deviation of the pre-operative pain interference t-scores. A patient was considered recovered after observing two consecutive MCID decreases in post-operative pain interference t-scores. A cox proportional hazards model stratified by preoperative PI score quartile was used to predict probability of patient recovery after adjusting for age, race, ethnicity, gender, and primary payor. The average and total potential savings for the cohort was derived using the number of patient-visits post-recovery.

**Results**: Two consecutive decreases of MCID measured by PI PROMIS t-scores were achieved 90 days post-operatively by 16%, 16%, 17%, and 23% of post-operative ankle patients in quartiles 1–4 respectively; post-operative foot patients achieved 18%, 11%, 20%, and 26% in quartiles 1–4 respectively. Days 30–60 recorded the greatest rate of improvement across quartiles with Q4 showing the greatest improvement. The least improvement occurred between days 90–120 across all quartiles. Patients were seen by the provider on average 2.84 times after achieving two consecutive MCID improvements, totaling $243.63 in expenses to institutional payers per patient. Avoiding these excess visits after MCID achievement could result in savings ranging from $38,593 to $122,002 for this given cohort.

**Conclusions**: PROMIS PI t-scores can be used to assess the need for ongoing follow up for surgical patients. If there is evidence that the patient has improved a clinically meaningful amount on two successive visits additionally follow-up may not be needed. Using PROs in clinical decision-making pathways as suggested in this research will eliminate unnecessary visits and save healthcare dollars and valuable resources.

## O26

### Identifying knowledge gaps in medical student education of patient-reported outcomes

#### Samuel Florentino, Suzanne Karan, Judith Baumhauer

##### University of Rochester School of Medicine and Dentistry, Rochester, USA

*Journal of Patient-Reported Outcomes* 2023, **7(1)**: O26

**Objective**: To examine contemporary knowledge of PROs among medical students; assess the impact formal education on PROs on medical student knowledge.

**Methods**: A 20-question IRB-approved survey was developed using validated methodology (expert review, cognitive interviews, pilot study). The survey was web-based and distributed by email to medical students at two accredited US allopathic medical schools. Secondly, to determine the effectiveness of formal education, 4th-year medical students at the host institution were invited to complete a survey inquiring about their knowledge of PROs before and 2 weeks after a PRO educational lecture.

**Results**: 137 medical students responded to the survey. The total response rate was 14.8% (137/925). 57% reported knowing "what a PRO is" while 54% correctly identified the definition of a PRO. < 8% received formal education regarding PROs; < 25% of respondents understood the need/value to incorporate PROs into patient care as identified by the Centers for Medicare & Medicaid Services. Respondents demonstrated positive attitudes towards PROs: 78% of responding medical students agreed PROs are important in delivering high-quality patient care; 70% would utilize PROs in future practice and 84% were interested in learning about PROs. Only 16% of respondents felt prepared to utilize PROs in a patient care setting. Among 121 responding 4th year medical students, 67% correctly answered the definition of PROs prior to formal education compared to 82% after education. 88% agreed PROs are an important component of providing high-quality care.

**Conclusions**: The results of this survey provide important insight into the current PRO knowledge gap for Medical Students. These deficiencies are accentuated by the low (< 20%) proportion of students who feel prepared to utilize PROs in a clinical setting. These gaps in the knowledge and preparedness to use PROs are a barrier to the delivery of high-quality care. Medical students agreed they would like to receive education on PROs (> 80%). Improvements in knowledge of PROs were identified with the implementation of formal education into medical education curriculum (25% greater correct response rate). Based on the current study, the implementation of formal education of PROs into medical education curriculum may help fill the knowledge and training gap.

## P28

### Dutch-Flemish translation of the Prosthetic Limb Users Survey of Mobility and planning the validation study

#### Charlotte Anné^1^, Fred de Laat^1^, Brian Hafner^2^, Dagmar Amtmann^2^, Jan Geertzen^3^, Leo Roorda^4^

##### ^1^Rehabilitation Centre Leijpark, Libra Rehabilitation Medicine & Audiology, Tilburg, Netherlands. ^2^University of Washington, Seattle, USA. ^3^Department of Rehabilitation Medicine, University Medical Center Groningen, Groningen, Netherlands. ^4^Amsterdam Rehabilitation Research Center | Reade, Amsterdam, Netherlands

*Journal of Patient-Reported Outcomes* 2023, **7(1)**: P28

**Objective**: The Prosthetic Limb Users Survey of Mobility (PLUS-M™) is an item bank for measuring patient-reported mobility in prosthetic limb users. The PLUS-M™ item bank can be applied as short forms or a computerized adaptive test. The PLUS-M™ was originally developed in English using methods similar to those used for Patient-Reported Outcomes Measurement Information System (PROMIS) item banks. We have translated the PLUS-M™ into Dutch-Flemish. Our next step is to validate the Dutch-Flemish PLUS-M™.

**Methods**: The Dutch-Flemish PLUS-M™ translation was performed by Functional Assessment of Chronic Illness Therapy multilingual translation (FACITtrans) using standardized methodology. The methodology was similar to that used for Dutch-Flemish translations of PROMIS item banks, except for the cognitive debriefings. Next, 300 Dutch and 300 Flemish adults with leg amputation will be invited to complete a survey twice, 6 weeks apart. This survey includes questions pertaining to patient characteristics, the Dutch-Flemish PLUS-M™ (whole item bank), the Dutch-Flemish PROMIS Physical Function v1.2 Short Form 4a, and the Dutch-Flemish PROMIS Mobility v2.0 item bank to study the validity (including Graded Response Model fit, cross-cultural and construct validity) of the PLUS-M™. Moreover, it contains a Global Rating of Change (GRCQ). Participants who report no changes in mobility on the GRCQ are included to study the test–retest reliability.

**Results**: Fourteen of the 42 questions required a separate Dutch and Flemish version. A key problem was the translation of the English word "walking". This word had to be translated differently in Dutch and Flemish. Data collection to assess the validity and test–retest reliability of the Dutch-Flemish PLUS-M™ is in progress. Results will be available at a later stage.

**Conclusions**: The PLUS-M™ v2.0 item bank was translated into Dutch and Flemish. The translation was performed FACITtrans using standardized methodology. The next step is to study the validity and reliability of the Dutch-Flemish PLUS-M™.

## O29

### Mental and social health of Dutch children and adolescents before and during the COVID-19 pandemic

#### Michiel Luijten, Maud van Muilekom, Hedy van Oers, Josjan Zijlmans, Tinca Polderman, Lotte Haverman

##### Amsterdam University Medical Center, Amsterdam, Netherlands

*Journal of Patient-Reported Outcomes* 2023, **7(1)**: O29

**Objective**: The COVID-19 pandemic outbreak and the restrictions profoundly impacted the mental and social health of children and adolescents. In this study we aim to assess the long-term impact on mental and social health of children in the Dutch general population on multiple measurement occasions during the COVID-19 pandemic and compare it to pre-COVID-19 reference data.

**Methods**: A representative general population sample of Dutch children aged 8–18 years was approached bi-annually (spring/autumn) starting in April 2020 until March 2022 (5 measurements total). They were asked to complete six self-reported PROMIS® questionnaires on mental and social health (Global Health, Peer Relationships, Anxiety, Depressive Symptoms, Anger, Sleep-Related Impairment) using computerized adaptive testing. For these questionnaires pre-COVID reference data were available (N = 2401). PROMIS T-scores between the various measurement occasions will be compared using linear mixed models.

**Results**: In total, 2401 (2018), 844 (April 2020), 746 (November 2020), 1128 (March 2021) and 1032 (November 2021) children and adolescents completed the questionnaires. Data of March 2022 is still in the collection phase. Preliminary results show decreased mental and social health at all COVID-19 pandemic measurement occasions compared to pre-COVID-19 data and no return to baseline (2018) outcomes. Results including the March 2022 measurement, which did not include any COVID-related restrictions, will be presented at the conference.

**Conclusions**: Thus far this study has shown a reduced mental and social health of children and adolescents during the COVID-19 pandemic. With the restrictions currently being lifted throughout the Netherlands and other parts of the world, investigating the long-term outcomes will provide us with valuable information to bring mental health(care) to the forefront of political decision making now and for future pandemics.

## O30

### Psychometric properties and inter-rater agreement of paediatric and parent-proxy PROMIS in children with sickle-cell disease

#### Michiel Luijten^1^, Maite Houwing^2^, Madieke Muntendam^2^, Maud van Muilekom^1^, Karin Fijnvandraat^1^, Hedy van Oers^1^, Lotte Haverman^1^, Marion Cnossen^2^

##### ^1^Amsterdam University Medical Center, Amsterdam, Netherlands. ^2^Erasmus Medical Center, Rotterdam, Netherlands

*Journal of Patient-Reported Outcomes* 2023, **7(1)**: O30

**Objective**: Sickle cell disease (SCD) has a profound impact on the physical, mental, and social health of affected children. The primary focus of care for SCD is preventive treatment for which monitoring of symptoms and health is required. As disease severity can vary substantially between individuals a generic approach to assessing health outcomes is required. In this study we aim to assess the psychometric properties of the generic pediatric and parent-proxy Patient-Reported Outcomes Measurement Information System (PROMIS) item banks in children with SCD.

**Methods**: Between December 2019 and December 2020, patients (5–17 years old) and their parents were approached to participate in the study at the Sophia Children’s Hospital and the Emma Children’s Hospital in the Netherlands. The following self-report and parent-proxy PROMIS domains were included in this study: Global Health, Cognitive Functioning, Pain Interference, Mobility, Fatigue, Anxiety, Anger, Depressive Symptoms and Peer Relationships. We assessed unidimensionality through confirmatory factory analysis, convergent validity with subscales from the Pediatric Quality of Life Inventory (related domains r > 0.5), discriminant validity (severe vs. less severe; Cohen’s d), reliability (Cronbach’s a and standard error of measurement (SEM)) and inter-rater reliability (intra-class correlation coefficients (ICC)) of the item banks.

**Results**: In total 102 patients and 102 caregivers completed all item banks, of which 72 were dyads. All item banks displayed sufficient unidimensionality and convergent validity. Discriminant validity was found for the expected domains of Global Health and Mobility (d > 0.5), however only parent-reports were able to discriminate on Fatigue (d = 1.02) and Pain Interference (d = 0.49). Reliability was acceptable (Cronbach’s a > 0.80, SEM < 0.44) for all item banks. Inter-rater reliability was strong for all item banks (ICC 0.60—0.78) except Peer Relationships (ICC = 0.47, r = 0.31) and Global Health (ICC = 0.26, r = 0.16).

**Conclusions**: The PROMIS item banks displayed sufficient psychometric properties for use in pediatric SCD care and research. Proxy reports seem viable as alternative to self-report forms of PROMIS.

## O32

### Impact of patient-reported supportive care needs on quality of life in ambulatory oncology

#### Vandana D. Sookdeo^1^, Akina Natori^2^, Tulay Koru-Sengul^3^, Matthew Schlumbrecht^4^, Carmen Calfa^2^, Jessica Maclntyre^5^, Roberto M. Benzo^6^, Patricia Moreno^3^, Tracy E. Crane^2^, Sophia F. Garcia^7^, Sara F. Pavlovic^6^, Frank J. Penedo^8^

##### ^1^Sylvester Comprehensive Cancer Center, Miller School of Medicine, University of Miami, Miami, USA. ^2^Division of Medical Oncology, Department of Medicine, Miller School of Medicine, University of Miami, Miami, USA. ^3^Department of Public Health Sciences, Miller School of Medicine, University of Miami, Miami, USA. ^4^Division of Gynecologic Oncology, Department of Obstetrics and Gynecology, Miller School of Medicine, University of Miami, Miami, USA. ^5^Sylvester Comprehensive Cancer Center, Miami, USA. ^6^Sylvester Comprehensive Cancer Center, University of Miami, Miami, USA. ^7^Department of Medical Social Sciences, Feinberg School of Medicine, Northwestern University, Chicago, USA. ^8^Sylvester Comprehensive Cancer Center, Psychology and Medicine, University of Miami, Miami, USA

*Journal of Patient-Reported Outcomes* 2023, **7(1)**: O32

**Objective**: Understanding patient needs arising from cancer/symptom burden and its correlation to quality of life is essential to promoting well-being in this patient population. This study assessed the relationship between self-reported supportive care needs and health-related quality of life (HRQoL) in ambulatory oncology.

**Methods**: Retrospective review was performed for patients assigned to My Wellness Check (MWC) questionnaire between October 2019–January 2022 at Sylvester Comprehensive Cancer Center. The MWC questionnaire uses a 13-item self-reported supportive care needs assessment along with the Functional-Assessment-of-Cancer-Therapy-General-7-item (FACT-G7) and administered via patient-portal. Supportive care needs assessed include help coping with illness, transportation, financial/insurance concerns, etc. Patients who completed both supportive care needs assessment and FACT-G7 were included in analysis. Descriptive statistics were calculated for patient demographics, clinical characteristics, endorsed supportive care needs and FACT-G7 score. Simple and multivariate linear regression were used to evaluate the association between number of supportive care needs and FACT-G7 scores, adjusting for demographics and clinical factors. All *p*-values were two-sided and < 0.05 was considered statistically significant.

**Results**: A total of 3386 patients were identified. The majority were White (88.8%), Hispanic (51.6%), living with partner (64.1%) and had active treatment (70.1%). 576 patients (17%) endorsed one or more practical needs; where 392 patients had 1 need (68%), 105 patients had 2 needs (18%), and 79 patients had 3 or more needs (14%). The number of supportive care needs was negatively associated with FACT-G7 score after adjusting demographic and clinical factors (β = − 2.06, 95% CI = − 2.29 to − 1.82, *p* < 0.0001). Also, poorer HRQoL was associated with being female, having no partner, having comorbidities, and receiving active treatment.

**Conclusions**: The results demonstrate that higher number of supportive care needs was negatively associated with HRQoL. Furthermore, lower HRQoL was identified in specific patient groups. Additional investigation is necessary to identify barriers in said groups to address/alleviate endorsed needs. Providing additional cancer support services and implementing translational resources–possibly with a precision-medicine focus, can prevent or address future needs. Employing a care coordination team approach tasked with continuous monitoring of patient needs will help improve the impact of PROs in the clinical setting, and therefore, improve our patients’ ability to live a fulfilling life.

## P33

### OPTIMAL time frame for admission of PROMs to total joint arthroplasty patients: a prospective study

#### Ahmad Alnasser, Puck Van Der Vet, Marilyn Heng

##### Massachusetts General Hospital, Boston, USA

*Journal of Patient-Reported Outcomes* 2023, **7(1)**: P33

**Objective**: To investigate differences in significance between administration of Patient-Reported Outcome Measures (PROMs) over the course of postoperative follow-up visits and determine at which point further PROMs questionnaires are redundant.

**Methods**: This prospective cohort study utilized patients presenting to Massachusetts General Hospital, Brigham and Women’s Hospital, and Newton Wellesley Hospital for either total hip or total knee arthroplasty. Patients were recruited via letter from their surgeon. The survey included the following questionnaires: PROMIS Physical Function Computer Adaptive Test (CAT), PROMIS Pain Interference CAT, and PROMIS SFv2.0 Ability to Participate in Social Roles and Activities. These surveys were administered at baseline, and 4–6 weeks, 3 months, and 6 months following surgery.

**Results**: 48 patients were included beginning August 2020. 45 (93.8%) received conservative treatment prior to surgery, with 38 (79.2%) having done physical therapy and 30 (62.5%) having cortisone injections. A paired T-test was conducted for different PROMs measurement T-scores compiled at each follow-up. For Physical Function CAT, there is significant difference (*p* < 0.0001) between the 4–6-week and 3-month follow-up period, but non-significant difference between 3- and 6-month follow-up (*p* = 0.053). Paired T-tests for Pain Interference CAT indicate significance between baseline and 4–6-week follow-up (*p* = 0.0014), between 4–6 week and 3-month follow-up (*p* < 0.0001) and between 3- and 6-month follow-up (*p* = 0.008). For PROMIS SFv2.0, there was less significance (*p* = 0.04) from the 3- to 6-month follow-up than there was from the baseline to 4–6-week follow-up (*p* = 0.00515) and from 4–6-weeks to the 3-month follow-up (*p* = 0.000016). The average return to work time was 2.3 ± 1.3 months for employed patients (n = 23). Graphically, the change in mean T-scores begins to plateau around the 6-month follow-up for both Physical Function and SFv2.0.

**Conclusions**: There is lack of significance in measuring PROMs Physical Function between 3-months and 6-months post-operation, and a lesser degree of significance for SFv2.0 in that period, which might suggest that the responses to those questionnaires begin plateauing in magnitude prior to 6 months post-operation, where patients may feel similar onwards. Pain Interference scores remained highly significant, suggesting they should continue being administered following 6-months. More investigation is required to determine if these PROMIS questionnaires are fully unnecessary long-term.

## P35

### Maximizing clinical use of PROMIS responses in population health

#### Stacy Schmitt^1^, Christopher Mull^2^, William Mauck^3^, Daniel Darveaux^4^, Timothy Maus^5^

##### ^1^Mayo Clinic Multidisciplinary Spine Center, Rochester, USA. ^2^Mayo Clinic Center for Digital Health, Rochester, USA. ^3^Mayo Clinic Division of Pain Medicine, Rochester, USA. ^4^Mayo Clinic Information Technology, Rochester, USA. ^5^Mayo Clinic Department of Radiology, Rochester, USA

*Journal of Patient-Reported Outcomes* 2023, **7(1)**: P35

**Objective**: Reducing patient and provider burden while creating meaningful clinical visualizations are critical outcomes in PRO development, implementation, and clinical use. The objective is to describe population health methodology and dashboards that are used to monitor and analyze PROMIS responses over a five-year cadence. This abstract is the third in a trilogy of PROMIS International Presentations describing the evolution of the Mayo Clinic Spine Care PROMIS CAT implementation, representing a population dataset of 2.4 M profile domains collected annually.

**Methods**: Through generous funding by the Gerstner Foundation, an innovative team of data experts and clinical leaders sought to create an industry-first population health registry and dashboard using EHR-derived individual patient data and PRO responses following image-guided interventional spine therapeutic procedures and surgeries. The Mayo Clinic Divisions of Pain Medicine and Musculoskeletal Spine Radiology identified > 90 image-guided procedures or surgeries to correlate Epic EHR patient demographic data with collected PROMIS t-scores.

**Results**: Epic EHR data stored in Epic Clarity data tables were extracted into Tableau dashboards which display population level data by procedural/surgical intervention, location, date, specialty, provider, patient demographics and PRO responses. More than 30,000 unique annual image-guided procedures and surgeries from 10 national locations are included in the dashboards. Clinicians can visualize individual patient responses in the Epic EHR and compare them to aggregate population health criterion. All data is filterable so clinicians can use the individual data to aid in clinical decision-making and patient counseling when dealing with subjective outcomes such as pain, physical function, and quality of life. Aggregate data serves as a clinical quality assurance tool, improves fiscal responsibility and opportunity, and advances clinical research.

**Conclusions**: Use of PROs in the clinical setting improves patient engagement, education, diagnosis, treatment, and continual monitoring of symptoms. Aggregated, population-based Epic EHR data allows creation of feedback loops of patient phenotypes and their response to care interventions. These feedback loops are necessary to create real-time care coordination for a chronic patient population and to validate current or publish future care algorithms for spine disease.

## O37

### Depression and suicide screening in orthopaedic clinics: balancing patient survey-response burden with best practices

#### Karma McKelvey^1^, Grant Dornan^2^, Caryn Lindsey^1^

##### ^1^Cedars-Sinai Medical Center, Los Angeles, USA. ^2^Dornan Statistical Consulting, Eagle, USA

*Journal of Patient-Reported Outcomes* 2023, **7(1)**: O37

**Objective**: Establish best practices for fulfilling Joint Commission requirements to screen patients annually for suicidality in the absence of a “gold standard” for suicidality screening in orthopaedic clinics. The organization where our clinics are housed added PHQ9 to our screening battery; however, the PHQ9 measures somatic symptoms (e.g., sleep disturbance, fatigue), which can manifest because of musculoskeletal conditions or injuries as well as depression. Item #9 of the PHQ9 is widely used in isolation to measure suicidal ideation, which is more strongly associated with suicide even than suicidal behavior (e.g., previous suicide attempt). We hypothesized that administering item #9 alongside existing screening for depressive symptoms (PROMIS-D) could more accurately identify patients in need of interventional support for depressive symptoms, including suicide risk, while minimizing patient survey-response burden.

**Methods**: Between February 2021 and January 2022, PHQ-9, PROMIS-D, PROMIS-PI and PROMIS-PF were concurrently administered during N = 3102 patient visits. Follow up by our LCSW was triggered by any of the following: (A) PROMIS-D > 59 and/or, (B) #9 > 0 and/or, (C) PHQ9 > 11.

We retrospectively considered an alternate, 2-pronged screening system using only items (A) and (B) and calculated negative predictive value (NPV) compared to the 3-pronged screen.

**Results**: The 2-pronged screen provided 93% NPV; positive predictive value was 100% as the 2-pronged alert rule was a subset of the 3-pronged alert rule. For the n = 153 visits where patients who would have triggered the 3-pronged screen would not have triggered the 2-pronged screen, mean ± SD PROMIS-PI and PROMIS-PF were 68 ± 6.5 and 33 ± 8.5, respectively.

**Conclusion**: For every 100 patients who do not trigger follow-up using the 2-pronged screen, we estimate that 7 would have triggered follow-up using the 3-pronged screen. In our cohort, such patients exhibited a high degree of pain and more limited physical function—while scoring < 60 on PROMIS-D and not reporting any suicidal ideation via PHQ-9 Item #9—suggesting health issues that may be more suitably addressed by their orthopaedic care provider (vs. LCSW). These data provide reasonable rationale for asking only item #9 alongside PROMIS-D to identify orthopaedic patients warranting follow-up for depressive symptoms inclusive of suicidality.

## P38

### Improving depression care in a large medical center with PROMIS universal screening

#### Kimberly Van Orden, Alexandra VanBergen, Kenneth Conner, Kathleen Fear, Benjamin Chapman, Daniel Maeng, Ian Cero, Caroline Silva, Judith Baumhauer

##### University of Rochester Medical Center, Rochester, USA

*Journal of Patient-Reported Outcomes* 2023, **7(1)**: P38

**Objectives**: The purpose of the project is to examine mortality outcomes for a cohort of patients in a large medical center in western New York who completed universal screening for depression using patient-reported outcomes (PROs) outside of behavioral health clinics. We hypothesize that patients who screen positive for depression are at increased risk for suicide and all-cause mortality, with patients screening as severely depressed at greatest risk. Prior research was conducted with patients receiving behavioral healthcare. Our study is novel because we are examining mortality outcomes in depressed patients not receiving mental health treatment—a group that accounts for the majority of suicide deaths in the United States.

**Methods**: Data come from the electronic medical records (EMRs) of 206,468 adult patients (age 18 or older) who completed depression screens (either PROMIS depression or PHQ-2/9) prior to healthcare visits from 2015 to 2018 as part of universal screening in several settings, including primary care, orthopedics, urology, and pain clinics. Depression T scores ≥ 60 (one standard deviation above average, considered “moderate” depression) were coded as positive (using a validated cross-walk between PROMIS and PHQ-9).

**Results**: Depression screens were positive in 14.2% of patients (n = 29,314), with more positive screens among younger versus older adults, women versus men, non-White versus White, and Hispanics versus non-Hispanics. These same sociodemographic indicators, as well as completing screening in primary care (versus specialty care) were also associated with greater likelihood of receiving depression treatment. All patients with positive screens, as well as an equal number of controls (non-depressed, selected at random) will be used to examine mortality outcomes via linkage with national death records. Analyses are underway to compare suicide and all-cause mortality between depressed and non-depressed patient samples, as well as reductions in risk associated with treatment.

**Conclusions**: Universal screening for depression is becoming standard of care in many clinical settings. Our results support the utility of universal depression screening for detecting untreated depression across diverse sociodemographic groups and clinical settings. Linking depression screening with data on premature mortality may promote population health strategies to identify and treat depression.

## O39

### PROMIS first choice in national core set of PROs and PROMs for medical specialty care

#### Caroline B Terwee^1,2^, Lotte Haverman^3,4,5^, Martijn AH Oude Voshaar^6,7,8^, Philip J van der Wees^9^, Anna JHM Beurskens^10^

##### ^1^Amsterdam UMC location Vrije Universiteit, Department of Epidemiology and Data Science, Amsterdam, Netherlands. ^2^Amsterdam Public Health research institute, Methodology, Amsterdam, Netherlands. ^3^Amsterdam University Medical Center, University of Amsterdam, Department of Child and Adolescent Psychiatry & Psychosocial Care, Emma Children’s Hospital, Amsterdam, Netherlands. ^4^Amsterdam Public Health research institute, Mental Health, Amsterdam, Netherlands. ^5^Amsterdam Reproduction and Development, Child Development, Amsterdam, Netherlands. ^6^National Health Care Institute (Zorginstituut Nederland), Diemen, Netherlands. ^7^Department of Medical Cell BioPhysics & TechMed Center, University of Twente, Enschede, Netherlands. ^8^Mediquest, Utrecht, Netherlands. ^9^Radboud University Medical Center, IQ Healthcare, Nijmegen, Netherlands. ^10^Maastricht University, School Caphri, Department Family Medicine, Maastricht, Netherlands

*Journal of Patient-Reported Outcomes* 2023, **7(1)**: O39

**Objective**: Implementation of PRO initiatives are hampered by the existence of many different patient-reported outcome measures (PROMs) and conflicting data collection standards. The aim of this project, initiated by the Dutch Ministry of Health, Welfare, and Sport (VWS), was to develop a consensus based standard set of generic PROs and PROMs to be implemented in Dutch daily medical specialty care across patient conditions.

**Methods**: VWS established a national working group of mandated representatives of all relevant umbrella organizations involved in Dutch medical specialty care together with PROM experts and patient organizations. A structured, consensus driven co-creation approach was used, including literature review, online meetings, feedback from national patient organizations and the umbrella organizations. The methodological experts defined criteria for PROM selection, which were authorized by the working group members. The ‘PROM-cycle’ methodology was used to select feasible, valid, and reliable PROMs to obtain domain scores for each of the generic PROs included in the set.

**Results**: A core set of eight generic PROs was endorsed across different levels of health: symptoms (pain & fatigue), functioning (physical function, social function/participation, mental function (anxiety & depression)), and overarching (quality of life & perceived overall health). The working group recommends assessing all eight PROs routinely in all patients as part of the daily workflow unless there are good reasons to deviate from this recommendation. The core set can be supplemented with disease-specific PROs and PROMs if needed. For each PRO a limited number of generic PROMs was endorsed. PROMIS short forms were selected as the preferred instruments for all PROs. To facilitate comparison of outcomes, the working group recommends reporting PRO scores of all PROMs on the PROMIS T-score metrics. Therefore, an online cross-walk platform will be developed. In the near future a core set will also be developed for children.

**Conclusions**: A core set of generic PROs was endorsed by relevant umbrella organizations involved in Dutch medical specialty care. Implementation of this set, with PROMIS measures as preferred instruments, will improve PROM use across medical specialty care, and support shared decision-making and healthcare improvement.

## P40

### PROMIS-D for both patients and orthopaedic surgeons: identifying barriers to addressing mental health with patients

#### Caryn Lindsey^1^, Karma McKelvey^1^, Grant Dornan^2^, Mark Vrahas^1^

##### ^1^Cedars-Sinai Department of Orthopaedic Surgery, Los Angeles, USA. ^2^Steadman Philippon Research Institute, Vail, USA

*Journal of Patient-Reported Outcomes* 2023, **7(1)**: P40

**Objective**: Use PROMIS-Depression (-D) scores to improve clinical practice by identifying patients who screen positive for severe depression. In 2018 we began alerting providers and requested documentation in patient files of any action taken. This effort achieved just 28% adherence.

**Methods**: Based on low physician-adherence, we aimed to devise ways to improve our practice using PROMIS-D scores. We aimed to understand physician barriers to determine whether an intervention or education program could increase adherence while further guiding development of our collaborative care model (CCM) including, but not dependent on, physician–patient intervention. Using principles of community-based participatory research, we developed a survey for orthopaedic physicians in our clinic to assess perceived barriers and facilitators to addressing depression with patients. The survey assessed stress immunity, depression, anxiety, and emotional intelligence while also exploring reported barriers and self-efficacy.

**Results**: Surveys indicated mild anxiety and depressive symptoms, moderate to high emotional intelligence, and high stress immunity. Most had received empathy skills training in medical school, reported moderate comfort addressing mental health concerns with patients, and cited lack of time and clinic-workflow interruption as barriers. The majority preferred consult and collaboration with our clinic’s on-premises licensed clinical social worker for patient-intervention.

**Conclusion**: Notification of patient severe depression-status alone was insufficient to persuade most physicians to intervene with depressed patients; in response, we developed a more comprehensive approach that took the onus off physicians in our clinic. However, our surgeons continue to report moderate comfort and self-efficacy in addressing mental health with patients while not actually intervening. It is important to support clinicians with self-serve resources or training aimed at improving self-efficacy and time management while supporting protective self-care strategies. Further, providing appropriate resources about depressive symptoms and how they can impact treatment for patients and nursing, or office staff could minimize stigma and maximize understanding and self-efficacy to discuss depressive symptoms with surgeons, especially as relates to the issue they are presenting for.

## P41

### From numbers to meaningful change: minimal important change by using PROMIS in fracture patients

#### Thymen Houwen^1^, Koen W.W. Lansink^2^, Mariska de Jongh^1^

##### ^1^Network Emergency care Brabant, Tilburg, Netherlands. ^2^Elisabeth-Tweesteden Ziekenhuis, Tilburg, Netherlands.

*Journal of Patient-Reported Outcomes* 2023, **7(1)**: P41

**Objective**: The Patient-Reported Outcomes Measurement Information System is increasingly being used in patients with an orthopedic fracture. Yet, minimal important change of PROMIS in patients with orthopedic fractures has only been addressed in a few studies with single fracture populations or heterogeneous general trauma populations. As the minimal important change (MIC) is important to interpret PROMIS-scores, our goal is to estimate the MIC for PROMIS physical function (PF), PROMIS pain interference (PI) and PROMIS ability to participate in social roles and activities in patients with an orthopedic fracture.

**Methods**: We conducted a retrospective study on patients ≥ 18 years receiving surgical or non-surgical care for orthopedic fractures between January and March 2022. Patients completed PROMIS PF V1.1, PROMIS PI V1.1 and PROMIS ability to participate in social roles and activities V2.0. At follow-up, patients completed three additional anchor questions evaluating patient-reported improvement on a seven-point rating scale. We used the mean change method and the ROC method to estimate the MIC value of all three separate PROMIS questionnaires.

**Results**: We included fifty patients with a mean age of 56 ± 12 years and thirty-one (62%) were female. Twenty-four (48%) patients were recovering from a surgical procedure. The mean change method showed a MIC value of 4.2 (n = 17) for PROMIS PF, 3.2 (n = 17) for PROMIS PI and 3.2 (n = 15) for PROMIS ability to participate in social roles and activities. The ROC method showed an optimal ROC cutoff point of 3.7 (n = 48) for the PROMIS PF questionnaire, 3.7 (n = 46) for PROMIS PI and 4.7 (n = 47) for PROMIS ability to participate in social roles and activities.

**Conclusions**: In the setting of orthopedic fractures, MIC values range in this study from 3.7 to 4.2 for PROMIS PF, 3.2 to 3.7 for PROMIS PI and 3.2 to 4.7 for PROMIS ability to participate in social roles and activities. MIC can be used in clinical practice for managing patient expectations; to inform on treatment results; and to assess if patients experience significant change. This in order to encourage patient centered care. Our results add to the growing knowledge on clinical importance by use of PROMIS.

## P43

### Validation of PROMIS profile-25 in a Swedish quality register study over a 3-year follow-up

#### John E. Chaplin^1^, Aina Danielsson^1^, Per-Mats Janarv^2^, Ebba Fridh^1^, Marie Askenberger^3^

##### ^1^Sahlgrenska Academy at Gothenburg University, Gothenburg, Sweden. ^2^Astrid Lindgren Children's Hospital, Karolinska Hospital, Stockholm, Sweden. ^3^Karolinska Institute, Stockholm, Sweden

*Journal of Patient-Reported Outcomes* 2023, **7(1)**: P43

**Objective**: To test the construct validity of PROMIS profile-25 against the standard KOOS-child (39 questions) in an orthopaedic register study (2015–2020). The Pediatric Orthopedic Quality Register (SPOQ) in Sweden collected patient responses at follow-up during scheduled hospital consultations after an acute severe knee injury.

**Methods**: Construct validity is indicated by a good and robust correlation between corresponding domains (PROMIS PF-Mobility and KOOS-child Sport/play; PROMIS Pain interference and KOOS-child Pain scales) over time (both repeated measures and independent samples). A stable relationship between the other domains would aid interpretation of both instruments. Spearman's correlation was used and similarity between correlations across time-points was assessed via a Z-score test. Internal reliability was compared using Cronbach's alpha.

**Results**: Of 368 children aged 9–14 years. (mean 12 y 8 m, 45% girls) at injury, treated between 2015 and 2019 there were 213 independent subjects (14 years, 44% girls) at year one follow-up, and 89 at year three follow-up (15 years, 40% girls). Sixty-three children responded at both years one (14 years, 55% girls) and three. PROMIS scores were within the normal range (40–60) at both follow-ups. KOOS-Child scores were within 80–100 points except Symptoms and QoL which were lower and did not enter the normal range at year three. Good correlations were found between the hypothesized variables at each time-point (0.767, 0.633; 0.755, 0.797). Relationships between other domains remained stable with the exception of KOOS-Symptoms (knee problems) which had a low and unstable correlation with PROMIS-mobility (0.319, 0.650). Internal reliability was good in all cases excluding KOOS-Symptoms (PROMIS: 0.769–0.920; KOOS: 0.845–0.959). KOOS-symptoms had poor internal reliability at each time point (alpha = 0.198, 0.484).

**Conclusions**: PROMIS profile-25 was validated against the KOOS-child and can be considered a useful instrument in this population of orthopedic patients. PROMIS adds social and psychological aspects to the PRO measurement not available in KOOS-child while at the same time providing equivalent measurement of mobility/sport and pain interference. KOOS-Symptoms requires further analysis to determine utility in follow-up. KOOS-symptoms has been noted previously to be more variable and less homogenous than the other KOOS subscales and this was found in this population.

## P44

### Psychometric properties of the Swedish PROMIS profile-29 in a population of patients with SSC

#### Dirk M. Wuttge^1^, Gunnel Sandqvist^1^, John E. Chaplin^2^

##### ^1^Lund University and Skåne University Hospital, Lund, Sweden. ^2^Institute of Clinical Sciences, Sahlgrenska Academy at Gothenburg University, Gothenburg, Sweden

*Journal of Patient-Reported Outcomes* 2023, **7(1)**: P44

**Objective**: To evaluate the floor and ceiling effects, external and internal reliability of the PROMIS profile-29 in a population of adults with SSc.

**Methods**: Consecutively enrolled, 18 years of age or older patients, fulfilling criteria for SSc, completed the first test during out-patient follow-up at a terciary rheumatology unit. The retest was completed within 2 weeks using questionnaires mailed to the patients at home. Test–retest reliability was analyzed with intraclass correlation coefficients (ICC) (> 0.70). Linear weighted kappa (Kw) coefficient was used to measure stability within test scores for the individual items in the PROMs. Internal reliability was tested at the first time-point with Cronbach’s alpha (0.70–0.95). Frequency distribution of patients scoring the lowest possible health (floor effects) and the best possible health (ceiling effect) were assessed.

**Results**: Forty-nine patients (86% female, 73% limited cutaneous SSc) with a mean disease duration of 11 years, mean SHAQ of 0.5 and mean modified Medsger Severity Score of 4.5 were enrolled. Test–retest reliability was good (ICC 0.78 to 0.94) with the exception of PROMIS-anxiety (ICC 0.67, CI = 0.37 to 0.83). Ceiling effects (> 15%) indicating best health were present in six PROMIS-29 domains (anxiety 33%, depression 35%, fatigue 16%, pain interference 29%, physical functioning 20% and ability to participate in social roles and activities 16%) Sleep disturbance was the only domain that did not show floor or ceiling effects.

**Conclusions**: The PROMIS profile-29 demonstrates good psychometric properties with good to acceptable external reliability and good internal reliability of each domain. Ceiling effects were found for most scales, and this should be considered with regard to the patient group and research question. The advantage of the PROMIS methodology is the flexibility in item selection. Longer short-forms might be added to the profile to increase sensitivity in the healthy range of the scale if this is important.

## P45

### Score thresholds for PROMIS measures: results of a virtual bookmarking study

#### Nan E. Rothrock^1^, Sandra A. Wilson^2^, Marilyn Heng^2^, Aleksandra Hodor^3^, Alexander Joeris^3^, Aaron J. Kaat^1^, Karma McKelvey^4^, Benjamin D. Schalet^1^, Mark Vrahas^4^

##### ^1^Northwestern University, Chicago, USA. ^2^Massachusetts General Hospital, Boston, USA. ^3^AO Foundation, Davos, Switzerland. ^4^Cedars-Sinai, Los Angeles, USA

*Journal of Patient-Reported Outcomes* 2023, **7(1)**: P45

**Objective**: PROMIS upper extremity function, physical function, and pain interference measures are increasingly utilized in orthopaedic research and patient care. In order to facilitate score interpretation, we aimed to adapt bookmarking, a standard setting methodology, to a virtual format and identify provisional score thresholds with this patient population.

**Methods**: We identified the components of bookmarking methods completed via in person focus groups that would be challenging to replicate in a virtual environment. We then adapted materials and procedures to address those challenges. We constructed vignettes comprised of 6 items and responses from the PROMIS v2.0 Upper Extremity function, v2.0 Physical Function, and v1.1 Pain Interference item banks using item parameters. For each domain, we created 7–9 vignettes, separated by 5 T-score points, each describing a fictional patient at various levels of health. Patients with a recent lower extremity fracture receiving orthopaedic care in an academic medical center were invited to participate. Participants independently placed bookmarks separating vignettes representing “within normal limits,” “mild,” “moderate,” and “severe” symptom/dysfunction. Participants then discussed bookmark placement for each domain until consensus was reached.

**Results**: New modifications to fit a virtual format included reducing the group meeting time and conducting individual pre-group meetings between all participants and study staff. New eligibility criteria for access and proficiency with video conference technology were added. Of the 8 patients enrolled in the study, 4 attended the focus group. All patients (age 29–59) had an ankle fracture that occurred 2–10 months prior to participation. All were able to complete the bookmarking activities independently and reach group consensus on thresholds for Upper Extremity (40, 30, 20), Physical Function (50, 40, 30), and Pain Interference (55, 65, 70). Additional virtual focus groups (1 with orthopaedic patients, 2 with clinicians) are scheduled in April and May 2022 to further explore the replicability of these findings.

**Conclusions**: Bookmarking methods for PROMIS measures can be utilized in a virtual environment. Thresholds vary by domain but are similar to previous in-person studies in different patient populations including those with rheumatoid arthritis and cancer.

## O46

### Clinical implementation and validation of PROMIS 2-item short forms for routine symptom monitoring in oncology

#### Laura M. Perry^1^, Sofia F. Garcia^1^, Michael A. Kallen^1^, JD Smith^2^, Susan Metzger^3^, September Cahue^1^, Nicola Lancki^1^, David Cella^1^

##### ^1^Northwestern University Feinberg School of Medicine, Chicago, USA. ^2^Spencer Fox Eccles School of Medicine at the University of Utah, Salt Lake City, USA. ^3^Northwestern Medicine, Chicago, USA

*Journal of Patient-Reported Outcomes* 2023, **7(1)**: O46

**Objective**: Routine symptom monitoring can improve cancer patient outcomes. We developed an electronic health record (EHR)-integrated program that screens patient symptoms (anxiety, depression, pain, fatigue, physical function) and supportive needs prior to oncology appointments. This program was developed to generate clinical alerts for elevated symptoms based on PROMIS computer adaptive test assessments. However, workflow limitations required that we shorten assessments to two items per symptom. Here, we will describe the implementation consequences of this modification and the clinical validity of shortened assessments.

**Methods**: We report available data from NU IMPACT, an ongoing implementation and effectiveness study across Northwestern Medicine oncology clinics. To evaluate implementation consequences of the assessment modification to 2-item short forms, we will report dimensions within the RE-AIM framework during the 6 months before and after this modification. Dimensions will include (1) proportion of eligible patients who received screeners (*level 1 Reach, Implementation fidelity*), (2) proportion of invited patients who completed screeners (*level 2 Reach*), and (3) proportion of eligible clinics that have sent out screeners (*Adoption*). To assess the clinical validity of 2-item PROMIS short forms, we will examine clinical alert rates and compare rates of healthcare utilization between patients who scored above and below clinical alert thresholds during 1-month post-baseline assessment.

**Results**: The NU IMPACT study began on 9/29/20; the shortened symptom screener was implemented on 9/29/21. 21,411 patients were invited to complete screeners during the 6 months before assessment modification; 21,957 patients were invited to complete screeners during the 6 months afterwards. The completion rate increased from 33% (7,114/21,411) to 37% (8,100/21,957) after implementing the shortened assessment version. Clinical alert rates remained stable or slightly increased from pre- to post-modification (anxiety: 3% vs. 3%; depression: 4% vs. 5%; fatigue: 1% vs. 3%; pain: 1% vs. 5%; physical function: 5% vs. 13%). We will present detailed additional results based on the RE-AIM framework and clinical validation analyses, utilizing updated datasets.

**Conclusion**: This project demonstrates (1) program changes that are responsive to clinical workflow needs can bolster implementation in healthcare systems, and (2) shortened PROMIS-based assessments can inform clinically valid symptom monitoring within standard oncology care.

## P47

### Realizing the promise of PROMIS: insights from one million responses

#### Nadine McCleary, Rachel C. Sisodia, Laurie Burnside

##### Mass General Brigham, Boston, USA

*Journal of Patient-Reported Outcomes* 2023, **7(1)**: P47

**Objective**: Mass General Brigham (MGB) has collected 1,028,869 PROMIS-10 surveys since 2015 and believes that to be the largest such repository in use. With extensive multi-specialty, longitudinal collection, MGB is positioned to be the real-world laboratory for this important, validated survey. We propose to share a descriptive analysis to include the following selected results.

**Methods**: The PROMIS 10 is included in most of MGB’s 181 questionnaire sets across specialties. Questionnaires are assigned/collected from patients via EPIC EHR using either a patient portal or clinic iPads. Since 10/1/21 all MGB PROMs appear in English plus 6 additional languages. Data sets for 5 broad specialty groupings were reviewed for the below summary results: Orthopedics, Radiation Oncology, Medical Oncology, Surgical Oncology, and all other specialties.

**Results**: Orthopedics has the most submission, at 294,318. Mental health scores indicate that 73.9% of Ortho responders fall within the normal limits for mental scores, 13.1% mild level of symptoms, 10.1% moderate response and 0.7% of total submissions were severe. The sociodemographic factors for Orthopedic patients completing the measure show that most respondents, 84.5%, were White, 5.4% Black, 2.9% Asian, 0.1% AIAN, 0.05% NHPI, and 0.77% identified as multiracial. Orthopedics Ethnicity data in the demonstrates that 86.5% were non-Hispanic, 4.8% Hispanic and 8.7% either unknown or declined to answer. The breakdown of Orthopedics respondents by age shows us that 50.8% were over 60, 19.7% were 50–59, 11.5% were 40–49, 8.8% were 30–39, 8.5% were between 18 and 29 and 0.7% were under the age of 18.

**Conclusions**: These data describe a PROMIS-10 data set of a magnitude adequate to allow MGB to perform deeper examinations, especially on health equity (REaL, SOGI and location), and mental health dimensions. Eventually MGB may evaluate the ability for PROMIS-10 scores to predict illness or recovery outcomes. This work will not only benefit MGB patients but can also be the basis for comparison and further research and publication by other health systems.

## P48

### PRO, one key to information driven care in the Region of Halland, Sweden

#### Markus Lingman^1^, John E. Chaplin^2^, Evalill Nilsson^3^, Lars Gustafsson^4^

##### ^1^Halmstad University, Halmstad, Sweden. ^2^Gothenburg University, Gothenburg, Sweden. ^3^Linnaeus University, Kalmar, Sweden. ^4^Halland Hospital, Halmstad, Sweden

*Journal of Patient-Reported Outcomes* 2023, **7(1)**: P48

**Objective**: Region Halland of Sweden performs well in both clinical results (quality) and cost efficiency. One important factor in this achievement is ‘Information driven care’. The region has a well-structured data warehouse that enables strategic analyses of the impact of care interventions for different patient groups and predictive clinical decision support, for example by weaving together patient-reported data with clinical data and using AI to identify risks to healthcare delivery.

The objective is to increase the ability to understand the overall benefits of healthcare for the patient from the patient's perspective, evaluate changes in healthcare and follow the development of the created benefit over time, set the patient-experienced benefit in relation to the overall efforts of the healthcare, and describe the patient benefit in an evidence-based and validated way.

**Methods**: A pilot project in 2022 has integrated PROMIS GH10v1.2 into Halland’s technology data ‘Platform24’. The design of the electronic questionnaire has been in association with PROMIS design guidelines. A pilot study in two medical areas (breast cancer and dialysis) using a smartphone app of PROMIS GH10v1.2 will collect data according to a schedule co-designed with the clinics to increase engagement and adjusted to local routines. The results, both per item and sub-domain t-scores, will be available instantly for clinical response. Methods of presenting results as time series and related to clinical change outcomes and care regime will be evaluated for comprehensibility and utility by clinical staff.

**Results**: Region Halland has now started the journey to introduce patient-reported health outcomes and experience of care. This pilot project will be evaluated prior to scaling up the usage of PROMIS and other PRO measures to the majority of the region’s patients in 2023. Other PROMIS short-forms and a move towards Computer-adapted-testing (CAT) are expected to be included in later phases.

**Conclusions**: The Director of Analysis at the Swedish Health and Social care inspectorate considers this to be one of the most important projects in Sweden 2022. Dr Evalill Nilsson, head of the e-health institute at Linnaeus University believes that item-banking and CAT will become a new national model for patient-reported health outcomes.

## P49

### Reliability of PROMIS CATs, short forms and legacy measurement instruments in patients undergoing total hip arthroplasty

#### Christel Braaksma^1^, Nienke Wolterbeek^1^, Remmelt Veen^1^, Yvette Pronk^2^, Rudolf Poolman^3,4^, Caroline Terwee^5^, Raymond Ostelo^5^

##### ^1^St. Antonius Hospital, Utrecht, Netherlands. ^2^ViaSana, Mill, Netherlands. ^3^Onze Lieve Vrouwe Gasthuis, Amsterdam, Netherlands. ^4^Leiden University Medical Center, Leiden, Netherlands. ^5^Amsterdam University Medical Center, Amsterdam, Netherlands

*Journal of Patient-Reported Outcomes* 2023, **7(1)**: P49

**Objective**: Currently used Patient-Reported Outcome Measures (PROMs) evaluating outcome of total hip arthroplasty (THA) have several problems regarding their validity and interpretation of scores. A relatively novel alternative is the Patient-Reported Outcomes Measurement Information System (PROMIS®) using Computerized Adaptive Test (CAT). In a CAT, it is thought that the questions presented are more relevant for patients, and patients need to complete less questions to get a reliable score. The goal of this study is to compare the test–retest reliability of the PROMIS CATs and short-forms to the legacy PROMS currently used in THA patients.

**Methods**: This prospective, multicenter study included adult patients on the waiting list for THA and patients who underwent THA in three district hospitals. Patients completed an online questionnaire twice with a 2-week interval, including two PROMIS CATs and four PROMIS short-forms (all assessing physical function and pain interference), PROMIS Pain Intensity single item, the Hip disability Osteoarthritis Outcome Score (HOOS), Oxford Hip Score (OHS), and Numeric Rating Scales (NRS) measuring pain during activity and in rest. Measurement precision (SEM), smallest detectable change (SDC) and the intra-class correlation coefficient (ICC) were calculated for all outcome measures to determine the reliability.

**Results**: 401 patients completed the questionnaires. Results showed a sufficient test–retest reliability (ICC 0.73–0.9) of all PROMs, PROMIS short forms and CATs except for the PROMIS CAT pain interference (ICC 0.67). The SEM of PROMIS instruments and legacy instruments ranged from 0.9 to 4.2, and from 3.1–10.4 respectively, across domains. Regarding pain intensity and pain interference, the PROMIS short forms demonstrated a better reliability and a smaller SDC compared to the legacy instruments and PROMIS CAT. PROMIS CAT and PROMIS short forms assessing physical functioning demonstrated an equal reliability and a smaller detectable change than the OHS.

**Conclusions**: The PROMIS CAT measuring physical functioning and the PROMIS short forms measuring physical functioning, pain intensity and pain interference are reliable measurement instruments, able to detect a smaller change than the legacy instruments. Therefore, these measurement instruments enable more accurate individual patient monitoring and improve the reliability of study results.

## P50

### Interpretability of Patient-Reported Outcome Measurement Information System (PROMIS) measures in rehabilitation populations: a systematic review

#### Rehab Alhasani^1^, Rebecca Ataman^2^, Line Enjalbert^2^, Alia Osman^2^, Henry Michael^2^, Adria Quigley^2^, Sara Ahmed^2^

##### ^1^Princess Nourah Bint Abdulrahman University, Riyadh, Saudi Arabia. ^2^McGill University, Montreal, Canada

*Journal of Patient-Reported Outcomes* 2023, **7(1)**: P50

**Objective**: To evaluate the interpretability of PROMIS measures by summarizing the available evidence among rehabilitation populations.

**Methods**: The systematic review was conducted according to the 2018 COnsensus-based Standards for the Selection of Health Measurement Instrument (COSMIN) guidelines. Seven electronic databases and two clinical trials registries were searched from 2004 to 2022. Two independent reviewers completed article selection and extracted study and patient characteristics. Measurement properties, interpretability and feasibility were synthesized, and the modified-GRADE approach was used to assess evidence quality. The results focus mainly on data interpretability.

**Results**: A total of 202 articles met the criteria and were included in the systematic review, including four rehabilitation populations. Preliminary results from 85 extracted articles showed that 56 PROMIS measures were identified. In addition, interpretability was reported among neurological population in 38% of articles; orthopedic in 39%; geriatric in 9%; and other chronic conditions in 14%. Fifty-five percent of articles evaluated interpretability for 30% physical function domain; 23% for pain; 13% for ability to participate in social roles, 10% for fatigue, and 10% for depression. Interpretability was evaluated using the (1) distribution-based method, and results showed a large range of estimates (PROMIS-Physical Function: smallest detectable change = 0.35–4; PROMIS-Pain Interference: smallest detectable change = 1.25–4.53); (2) anchor-based method and results showed an extensive range of estimates (PROMIS-Fatigue: minimal important change = 1.17–4.24 T-score; PROMIS-Physical Function: minimal important change = 3.25–11.70 T-score); (3) responsiveness and results showed a moderate to large effect sizes for PROMIS-Physical Function, PROMIS-Pain Interference, PROMIS-Fatigue, and PROMIS-Depression. Final results will be presented.

**Conclusions**: There is strong evidence for the interpretability of PROMIS-Physical Function among the orthopedic population. Limited information on the smallest detectable change of PROMIS measures. Further research is needed to evaluate interpretability in non-orthopedic populations and psychosocial domains.

## O51

### Impact of measurement precision of PROMIS tools on statistical power

#### Felix Fischer

##### Charité Universitätsmedizin Berlin, Berlin, Germany. PROMIS National Center Germany, Berlin, Germany

*Journal of Patient-Reported Outcomes* 2023, **7(1)**: O51

**Objective**: One of the main advantages of patient-reported outcome measures (PROMs) based on item-response theory such as PROMIS tools is that measurement precision of the individual test score is transparent. Score precision varies across the continuum of the target construct as well as between types of measures such as fixed short forms and computer-adaptive tests. A flexible technique to account for measurement error in statistical analysis of PROMs is plausible value estimation. The aim of this study is to investigate how measurement precision of PROMIS tools affects statistical power.

**Methods**: We simulated 60,000 studies, each comparing 2 groups, and systematically varied mean theta score (− 2 to 4), theta group difference (0 to 1) and group size (10 to 500). In each study, we randomly sampled true theta values and used those to simulate item-level data based on the PROMIS Anxiety itembank. The resulting item responses were scores using 3 different PROMIS measures (PROMIS Anxiety SF4a, SF8a, CAT). The simulated data was analyzed in a regression framework either directly using EAP estimates, ignoring measurement error, or accounting for measurement error by imputing 25 sets of plausible values and pooling results. We then assessed power (probability for *p* < 0.05 if H1 is true) depending on study characteristics.

**Results**: We observed significant (*p* < 0.05) effects of all simulation parameters (n, theta score, effect size, instrument, analysis type) on statistical power. Given constant sample size and effect size, we observed that power to detect group differences was higher in the medium theta range compared to the limits and that CATs had higher power than 8 and 4 item short forms. Analyzing plausible values resulted in less power compared to analysis of raw EAP estimates, but confidence intervals of effect estimates covered true effect more often.

**Conclusions**: Sample size planning using PROMIS tools as outcome requires careful assessment not only of expected effect size, but also of expected theta scores and measurement error of PROMIS tools used. Simulation based approaches can help to inform proper sample size estimation for studies using PROMIS tools as outcomes.

## O52

### Cross-European validation of PROMIS Profile 29 summary scores

#### Felix Fischer

##### Charité Universitätsmedizin Berlin, Berlin, Germany. PROMIS National Center Germany, Berlin, Germany

*Journal of Patient-Reported Outcomes* 2023, **7(1)**: O52

**Objective**: The PROMIS Profile 29 can be used to calculate physical and mental health summary scores. As the underlying statistical model was developed based on US data exclusively, we aimed to investigate applicability of this scoring algorithm in samples collected across Europe.

**Methods**: We fitted the proposed confirmatory factor analysis (CFA) model in PROMIS Profile 29 data from 3 general population samples collected in the UK (n = 1509), France (n = 1501) and Germany (n = 1502) using a Bayesian framework. Bayesian CFA allows to take prior information on model parameters into account during model fitting. We fitted three models per sample, resembling a continuum between full replication of the proposed model (using strongly informative priors) and full re-estimation of the model in new data (weakly informative priors). We compared measurement model parameters (loadings and factor correlations) across the different models and investigated agreement between resulting summary scores.

**Results**: Even under weakly informative priors, the measurement model could be largely replicated, but we observed considerable differences in some factor loadings (up to 0.2 points). These differences point to a stronger influence of pain to physical health and weaker to mental health in the UK. Also, ability to participate in social roles was stronger associated with mental health than with physical health in German samples. Correlation between physical and mental health was consistently observed to be smaller in European samples. Summary scores calculated by the US scoring algorithm and those from European models correlated strongly (r > 0.90).

**Conclusions**: European data suggests a slightly different composition of summary scores for each country. It remains unclear, whether those differences are due to sampling error or actual cultural differences. Bayesian modeling offers a long-term perspective to combine model parameter from different studies.

## P53

### Test–retest reliability and minimal detectable change of PROMIS CATs in patients receiving physical therapy

#### Erik-Jan Haan^1,2^, Caroline Terwee^3,2^, Harriet Wittink^1^, Philip Van der Wees^4^, Raymond Ostelo^3,5^, Henri Kiers^1^

##### ^1^Research Group Lifestyle and Health, HU University of Applied Sciences, Utrecht, Netherlands. ^2^Amsterdam Public Health Research Institute, Amsterdam, Netherlands. ^3^Amsterdam University Medical Center, Vrije Universiteit Amsterdam, Department of Epidemiology and Data Science, Amsterdam, Netherlands. ^4^IQ Healthcare and Rehabilitation, Radboud University Medical Center, Radboud Institute for Health Sciences, Nijmegen, Netherlands. ^5^Department of Health Sciences, VU University, and Amsterdam Movement Sciences, Amsterdam, Netherlands

*Journal of Patient-Reported Outcomes* 2023, **7(1)**: P53

**Objective**: The aim of this study is to examine the test–retest reliability and minimal detectable change (MDC) of the Dutch-Flemish Patient-Reported Outcomes Measurement Information System Physical functioning v1.2 (PROMIS-PF), Upper extremity v2.0 (PROMIS-UE) and Pain interference v1.1 (PROMIS-PI) item bank administered as Computerized Adaptive Test (CAT) in patients receiving physical therapy.

**Methods**: Adult (> 18 years) patients with musculoskeletal disorders of the lower back, neck, or upper extremity from 13 primary care physical therapy clinics were included. At admission (T1), a questionnaire with demographic and clinical characteristics and the PROMIS CATs were administered. After 3 to 14 days (T2), the PROMIS CATs and anchor questions that measure change on the construct were administered. Test–retest reliability of the PROMIS CATs was assessed by calculating the Intraclass Correlation Coefficient (ICC_2,1_) and minimal detectable change (MDC) for “unchanged” patients between T1 and T2. The MDC, based on Item Response Theory, of each PROMIS CAT domain varies per patient and was calculated using the following formula: 1.96*√(SE_1_^2^ + SE_2_^2^) whereby SE_1_ is the individual’s IRT estimated standard error of the T-score at baseline and SE_2_ at the 3 to 14 days (T2) measurement. A mean MDC of each domain was subsequently calculated for the whole group.

**Results**: Patients with low back or neck pain (n = 55, PROMIS-PF), upper extremity pain (n = 37, PROMIS-UE), and either low back, neck, or upper extremity pain (n = 81, PROMIS-PI), completed the PROMIS CATs at T1 and T2 and were “unchanged” on the relevant domain’s anchor question at T2.

The mean (SD) T-score at T1 was 43.9 (5.2) for PF, 36.4 (7.7) for UE and 58.7 (5.5) for PI. The ICC (95% CI) of PROMIS CAT T-scores were 0.78 (0.65–0.86) for PF, 0.88 (0.78–0.94) for UE and 0.68 (0.54–0.78) for PI. The MDC (min–max) was 5.45 (4.2–6.1), 5.89 (4.6–8.6) and 4.94 (2.8–11.4) T-score points, respectively.

**Conclusions**: In patients with musculoskeletal disorders receiving physical therapy, PROMIS CATs showed sufficient test–retest reliability, and MDC values that are comparable to previous research. Further research is needed to examine the responsiveness and minimal important change of PROMIS CATs in this population.

## O54

### Reliability of PROMIS CATs, shortforms and legacy measurement instruments in patients undergoing total knee arthroplasty

#### Christel Braaksma^1^, Nienke Wolterbeek^1^, Remmelt Veen^1^, Yvette Pronk^2^, Rudolf Poolman^3,4^, Caroline Terwee^5^, Raymond Ostelo^5^

##### ^1^St. Antonius Hospital, Utrecht, Netherlands. ^2^ViaSana, Mill, Netherlands. ^3^Onze Lieve Vrouwe Gasthuis, Amsterdam, Netherlands. ^4^Leiden University Medical Center, Leiden, Netherlands. ^5^Amsterdam UMC, Amsterdam, Netherlands

*Journal of Patient-Reported Outcomes* 2023, **7(1)**: O54

**Objective**: Patient Reported Outcome Measures (PROMs) are used to evaluate the results of total knee arthroplasty (TKA). The PROMs currently used, have several problems regarding their quality and interpretation of scores. A relatively novel alternative is the Patient-Reported Outcomes Measurement Information System (PROMIS®) using Computerized Adaptive Test (CAT). In a CAT, it is thought that the questions presented are more relevant for patients and patients need to complete less questions to get a reliable score. The goal of this study is to compare the test–retest reliability of the PROMIS CATs and short-forms to the legacy PROMS currently used in TKA patients.

**Methods**: This prospective, multicenter study included adult patients on the waiting list for THA and patients who underwent THA in three district hospitals. Patients completed an online questionnaire twice with a 2-week interval, including two PROMIS CATs and four PROMIS short-forms (all assessing physical function and pain interference), PROMIS Pain Intensity single item, the Knee disability Osteoarthritis Outcome Score (KOOS), Oxford Knee Score (OKS), and Numeric Rating Scales (NRS) measuring pain during activity and in rest. Measurement precision (SEM), smallest detectable change (SDC) and the intra-class correlation coefficient (ICC) were calculated for all outcome measures to determine the reliability.

**Results**: 391 patients completed the questionnaires. Results showed a sufficient test–retest reliability (ICC 0.7–0.9) of all PROMs, PROMIS short forms and CATs except for the PROMIS CAT pain interference and NRS pain in rest (respectively ICC 0.64 and 0.68). The SEM of PROMIS instruments and legacy instruments ranged from 1–3.85, and from 10.6–14.47 respectively, across domains. The SDC of PROMIS instruments and legacy instruments ranged from 2.8–10.7, and from 29–40 respectively, across domains.

**Conclusions**: The PROMIS CAT and shortforms measuring physical functioning and pain interference demonstrated an equal reliability, and better measurement precision than the legacy instruments. Therefore, these measurement instruments enable more accurate individual patient monitoring and improve the reliability of study results.

## O55

### Evaluating systematic implementation of PROMIS-10 in ambulatory oncology: REALLS analysis of 10,691 patient responses

#### Michael Manni^1^, Jessica Cleveland^1^, Sarah Whittaker^1^, Sunyi Zhang^1^, Brittany Black^2^, Ellana Haakenstad^1^, Michael Hassett^1^, Nadine Jackson McCleary^1^

##### ^1^Dana-Farber Cancer Institute, Boston, Massachusetts, USA. ^2^Mass General Brigham, Boston, Massachusetts, USA

*Journal of Patient-Reported Outcomes* 2023, **7(1)**: O55

**Objective**: Since January 2018, the Patient Reported Data (PRD) Program at Dana-Farber Cancer Institute (DFCI) has routinely screened new patients using the Patient-Reported Outcomes Measurement Information System-Global Health questionnaire (PROMIS-10). In this analysis, we calculate and stratify response rates (RR), Global Physical Health (GPH) and Global Mental Health (GMH) scores by race, ethnicity, age, language, location, and sexual orientation and gender identity (REALLS) to inform interventions to improve outcomes.

**Methods**: Patients are prompted to complete an electronic health record (EHR)-integrated PROMIS-10, available in English or Spanish on any internet-enabled device or tablets provided in clinic. Clinicians access GPH/GMH scores within the EHR; Scores below the population mean (GPH < 50, GMH < 48) are highlighted to prompt intervention. In our analysis we stratify RR, GPH and GMH by REALLS. We focus on responses collected during 2021 to limit impacts of non-standardized questionnaire response collection during the height of the COVID-19 pandemic.

**Results**: Between 01/01/2021 and 12/31/2021, 10,691/27,881 (38%) eligible patients completed PROMIS-10. English-speaking patients had RR of 47% versus RR of 26% for Spanish-speaking patients. 46% of English-speaking responders (4792/10395) had GPH and 38% (3926/10395) had GMH below the population mean vs 70% of Spanish-speaking responders (53/76) having GPH and 61% (46/73) having GMH below the population mean. Patients self-identifying as White have RR of 42%, with 46% of White responders (4346/9474) having GPH below the population mean and 37% (3542/9597) GMH below population mean. Asian, Black, American Indian, or Other (BIPOC) patients had a RR of 34%, with 54% (408/761) of BIPOC responders having GPH below the population mean and 42% (310/761) having GMH below population mean. Differences are also observed when stratifying RR, GPH, and GMH by ethnicity, age, location, sexual orientation, and gender identity.

**Conclusions**: We have demonstrated the ability to gather patient-reported GPH and GMH data for a substantial proportion of patients in routine care using an EHR-integrated PROMIS-10. Further work is needed to understand if observed differences in RR, GPH and GMH are disparities. Results of this analysis will inform efforts to improve response rates and the implementation of tools to bolster timely clinician response to PROMIS.

## P58

### Translation and linguistic validation of PROMISnq Physical Function-MS15a & PROMIS Fatigue-MS8a for use in India/Malaysia

#### Emily Parks-Vernizzi^1^, Barbara Perez^1^, Emna Maksud^2^, Jiyoung Son^2^, Benjamin Arnold^1^, Helena Correia^2^

##### ^1^FACITtrans, Ponte Vedra, Florida, USA. ^2^Northwestern University, Feinberg School of Medicine, Department of Medical Social Sciences, Evanston, Illinois, USA

*Journal of Patient-Reported Outcomes* 2023, **7(1)**: P58

**Objective**: The purpose of this study was to translate and linguistically validate the PROMISnq SF v2.0—Physical Function—MS 15a and PROMIS SF v1.0—Fatigue—MS 8a in Assamese, Bengali, Gujarati, Hindi, Kannada, Malayalam, Marathi, Tamil, and Telugu; and report on challenges and solutions encountered during the process.

**Methods**: We translated 15 adult PROMIS Physical Function items and eight adult PROMIS Fatigue items using the FACIT methodology, which is a standardized iterative process of forward- and back-translation, expert review, harmonization, and cognitive interviewing. The translation team consisted of native speaking linguists of each language from India and Malaysia (Tamil only). For each language version, five native-speaking participants from the general population evaluated the relevance, comprehensibility, and appropriateness of the translated items. We conducted qualitative analysis of cognitive interviews to evaluate the linguistic equivalence of each translated item and provide insight into the relevance of the concepts.

**Results**: The sample consisted of fifty adults (26 women, 24 men) who were native speakers of each respective language from the general population and born in India or Malaysia (Tamil only). Cognitive interviews revealed two Fatigue concepts required revisions: “social activities” (Hindi) and “push yourself” (Telugu). Five Physical Function concepts required revisions: “floor” (Gujarati) and “limit,” “shoelaces,” “toilet” and “vacuuming” (Tamil, both India and Malaysia respondents). The revisions resolved particular cultural and linguistic issues apparent in respondent commentary for each language.

**Conclusions**: The Assamese, Bengali, Gujarati, Hindi, Kannada, Malayalam, Marathi, Tamil, and Telugu PROMISnq SF v2.0—Physical Function—MS 15a and PROMIS SF v1.0—Fatigue—MS 8a are considered conceptually equivalent to the English and can be used for patient-reported outcomes assessment in international research studies, clinical trials and practice.

## P59

### Patient-reported measurements: implementation and symptom improvement on acute inpatient psychiatric units

#### Savannah Layfield^1^, Samantha Wong^1^, Fernando Rodriguez-Villa^1,2^, Steven Gelda^1,2^, Eliot Gelwan^1,2^, Jane Eisen^1,2^, Kerry Ressler^1,2^, Agustin Yip^1,2^

##### ^1^Division of Depression and Anxiety, McLean Hospital, Belmont, USA. ^2^Department of Psychiatry, Harvard Medical School, Boston, USA

*Journal of Patient-Reported Outcomes* 2023, **7(1)**: P59

**Objective**: Patent-reported measures have long supported personalized patient care and outcome monitoring at McLean Hospital. These efforts expanded to the Division of Depression & Anxiety Disorders (DDAD) inpatient units in 2019, where patient-reports collected at admission and discharge are provided to clinicians to inform and monitor care. The present work utilizes the aggregate data collected on DDAD units to establish feasibility of collection on inpatient psychiatric units and characterizes treatment outcomes including symptom severity, comorbidity prevalence, demographics, and symptom improvement.

**Methods**: The preliminary sample consisted of 501 adults between the ages of 17 and 75, who completed PROMs as part of an ongoing QI/QC project. Pending analysis will examine collections to date at time of presentation. QIDS-SR16, GAD-7, and BASIS24 were administered 48-h from admission and discharge. The MSI-BPD, PCL-5, and a substance use screener assessed potential comorbidities at admission. Statistical analyses were performed with RStudio 4.1.1, using summary statistics and principal component analyses within base and stats (prcomp function) packages respectively.

**Results**: At admission, patients predominantly endorsed severe depressive symptom severity (33.3%) on the QIDS, severe anxiety symptom severity (50.3%) on the GAD-7, and having some thoughts of ending their life (71.5%) on the BASIS24. Additionally, 28.3% of patients screened positive for borderline personality disorder on the MSI-BPD, 25.0% screened positive for PTSD on the PCL-5, and 48.7% reported hazardous alcohol use on the AUDIT-C. Average functioning assessed by the BASIS24 (M = 37.9, SD = 14.1) indicated mild to moderate impairment. The GAD-7 scores (t(374) = 24.17, *p* < 0.001), the QIDS-SR16 scores (t(373) = 24.80, *p* < 0.001), and BASIS24 scores (t(385) = 24.76, *p* < 0.001) significantly decreased from admission to discharge. At discharge, most patients (53.7%) reported no thoughts of ending their life on the BASIS24. Univariate and principal component analyses suggest missingness of discharge assessments was unrelated to symptom severity.

**Conclusions**: This ongoing work suggests systematic self-assessment of psychiatric symptoms, with screeners evaluating modifiers of treatment effect, may be an efficient and valuable tool in acute psychiatric settings for assessing and monitoring care. The resultant dataset provides a glimpse into the psychiatric symptoms and comorbidities encountered in an inpatient setting and observed improvement with treatment.

## P61

### Patient-Reported Outcomes Education Series-Department of Orthopedics

#### Morgan Gulley, Matthew Watson, Catherine Olinger

##### University of Iowa Hospitals and Clinics, Iowa City, USA

*Journal of Patient-Reported Outcomes* 2023, **7(1)**: P61

**Objective**: By identifying a multidisciplinary approach to optimize workflow for both patients and clinicians and understanding the use of PROs in modern healthcare, we aimed to increase rates of completed generic and service specific PROs through the creation of an educational tool for the implementation of Patient-Reported Outcomes (PROs) as a standard for orthopedic clinical care.

**Methods**: Clinical and non-clinical staff within the University of Iowa Hospitals and Clinics (UIHC) Department of Orthopedics and Rehabilitation were provided an educational series (EDS). They were asked to complete a simple survey about their involvement and understanding of PROs for clinical care prior to being given a presentation. The presentation covered differences between validated PROs and regularly required health history questionnaires as well as additional topics on how the PROs are being used by insurance payment plans, for registries, and at UIHC, including retrospective research, monthly capture rates, and clinical decision-making. Questionnaire delivery workflows such as how to open the PROs for patients and assist patients when questions arise were also covered. A post-EDS survey was sent out to staff in an effort to assess new/additional understanding. After completion of the EDS, PRO capture rates from the 3 months prior and the 3 months after will be compared.

**Results**: Pre-EDS survey results showed that 41% of staff know that there is a way for providers to be informed that PROs are completed, 42% of staff knew that PROs were used for clinical decision-making, and 20% of staff thought PROs were an 8 out of 10 for importance in clinic. Capture rates from pre-EDS to during EDS showed an increase of about 10% for all PROs given during clinic. Post-EDS results showed an increase in all three areas from pre-EDS (64%, 46%, and 31% respectively).

**Conclusion**: Education and involvement of additional clinical and non-clinical staff and/or care teams helps drive higher PRO capture rates. By providing all clinic staff with the necessary information and workflow guidance, we were able to break down certain barriers to PRO collection.

## O62

### Psychometric analysis and validation of five new banks to measure mindfulness

#### Kathryn Jackson^1^, Benjamin Schalet^1^, Seung Choi^1^, Lan Yu^2^, Bruriah Horowitz^1^, Nathan Dodds^2^, Christina Sauer^1^, Natalia Morone^3^, Paul Pilkonis^2^, Carol Greco^2^, David Victorson^1^

##### ^1^Northwestern University, Chicago, USA. ^2^University of Pittsburgh, Pittsburgh, USA. ^3^Boston Medical Center, Boston, USA

*Journal of Patient-Reported Outcomes* 2023, **7(1)**: O62

**Objective**: Mindfulness-based interventions (MBIs) have a prominent place in public health and clinical research, but no comprehensive self-report measures of mindfulness exist. Using PROMIS development procedures, we created new mindfulness item banks for use by clinicians and researchers that target the full range of mindfulness experience, from entirely naive to expert practitioner. Here we report on the psychometric development and initial validation of these banks and their short-forms (SFs).

**Methods**: After completing qualitative research steps, we tested 216 candidate items with a general population internet panel (n = 4188) and a panel of meditation practitioners and teachers (n = 502). We conducted psychometric analyses (factor analysis, DIF, local dependency) to identify and finalize the banks. For validation, we administered the new mindfulness measures and legacy measures to 300 adult participants in ongoing MBI programs across the US. Construct and discriminant validity were assessed using correlations with legacy measures and PROMIS-29 measures, respectively. Participants were grouped by their level of mindfulness meditation experience in two ways: ever vs. never practiced, and current vs. previous vs. never practiced. Known groups validity was assessed using one-way Analysis of Variance, modelling difference in mindfulness T-score by meditation experience.

**Results**: Psychometric analyses resulted in 65 item deletions, leaving five domains: Allowance, Boundlessness, Insight, Openheartedness, and Presence. The banks were individually calibrated under the graded response model and centered on the general population. For each bank, 8-item SFs were selected based on CAT simulations and content diversity. In the validation sample,138 (46%) participants meditated currently. This group scored somewhat higher than non-meditators on Openheartedness (Cohen’s d = 0.45–0.48), and much higher on the other four mindfulness domains (d = 0.89–1.04). Significant increases in mindfulness were found with increasing meditation experience (*p* < 0.001 for all domains). The mindfulness measures were generally highly correlated with conceptually similar legacy measures (e.g., Presence and FFMQ-Observe (r = 0.76); Allowance and FFMQ-Non-reactivity (r = 0.72); Boundlessness and NADA-T(r = 0.79)). Correlations with PROMIS physical function and pain interference was not significantly different from zero, supporting discriminant validity.

**Conclusion**: The new mindfulness measures distinguish known groups and show convergent and discriminant validity. Next steps include assessing responsiveness to change and submission for PROMIS adoption.

## O63

### The association of granular, patient-level social determinant of health factors on presenting PROMIS Global-10 scores

#### David N. Bernstein^1,2^, Amanda Lans^3,4^, Aditya V. Karhade^1^, Marilyn Heng^3^, Rudolf W. Poolman^2^, Joseph H. Schwab^3^, Daniel G. Tobert^3^

##### ^1^Harvard Combined Orthopaedic Residency Program (HCORP)/Massachusetts General Hospital, Boston, USA. ^2^Leiden University Medical Center, Leiden, Netherlands. ^3^Massachusetts General Hospital, Boston, USA. ^4^University Medical Center Utrecht, Utrecht, Netherlands

*Journal of Patient-Reported Outcomes* 2023, **7(1)**: O63

**Objective**: Routinely collected sociodemographic characteristics (e.g., race and insurance type) and geography-based SDoH measures (e.g., Area Deprivation Index [ADI]) are associated with health disparities. However, the impact of patient-level SDoH factors (e.g., housing status) is not as well documented. We aimed to assess granular SDoH characteristics on presenting physical function and mental health, as measured by the PROMIS Global-10.

**Methods**: New orthopaedic patients at a single Level 1 trauma center were identified from 3/2018–12/2020. Included patients completed the PROMIS Global-10 as part of routine clinical care and visited their primary care physician (PCP) and completed a series of specific SDoH questions. The SDoH questions focused on transportation, housing, employment, and ability to pay for medications. Demographic and clinical information was abstracted from the electronic medical record. Two multivariable linear regression models were created to determine which “traditional” metrics and patient-specific SDoH factors were associated with worse presenting physical and mental health symptoms at presentation. The concept of the minimal clinically important difference (MCID) was used to denote clinical significance to our findings, while *p* < 0.05 was statistically significant.

**Results**: A total of 9,057 patients were included. Lack of reliable transportation to attend doctor visits or pick up medications (β = − 4.52 [95% CI: − 5.45 to − 3.59], *p* < 0.001), trouble paying for medications (β = − 4.55 [95% CI: − 5.55 to − 3.54], *p* < 0.001), Medicaid (β = − 5.81 [95% CI: − 6.41 to − 5.20], *p* < 0.001), and Workers’ Compensation (β = − 5.99 [95% CI: − 7.65 to − 4.34], *p* < 0.001) were associated with clinically worse presenting function. Trouble paying for medications (β = − 6.01 [95% CI: − 7.10 to − 4.92], *p* < 0.001), Medicaid (β = − 5.35 [95% CI: − 6.00 to − 4.69], *p* < 0.001), and Workers’ Compensation (β = − 6.07 [95% CI: − 7.86 to − 4.28], *p* < 0.001) were associated with clinically worse presenting mental health.

**Conclusions**: Transportation issues and financial hardship are associated with worse physical function and mental health. However, when accounting for these factors, Medicaid and Workers’ Compensation remains associated with worse symptom presentation, suggesting they capture other constructs. Interventions to decrease health disparities should not just focus on sociodemographic variables (e.g., insurance type) but these tangible patient-specific SDoH characteristics as well.

## O64

### Cross-validating the KOOS-12-Function to PROMIS Physical Function Link

#### Aaron Kaat^1^, Benjamin Schalet^1^, Patricia Franklin^1^, Xiaodan Tang^1^, Nan Rothrock^1^, Marilyn Heng^2^

##### ^1^Northwestern University, Chicago, USA. ^2^Massachusetts General Hospital, Boston, USA

*Journal of Patient-Reported Outcomes* 2023, **7(1)**: O64

**Objective**: The 12-item Knee Injury and Osteoarthritis Outcome Score (KOOS-12) short form is a frequently used patient-reported outcome measures for individuals with various knee injuries. The 4-item KOOS-12-Function score has previously been linked to PROMIS Physical Function (PF) so that scores can be converted from one measure to the other. This linkage was developed with total knee arthroplasty patients. To examine its generalizability, we sought to cross-validate this linkage in a large sample of older adults with arthritis.

**Methods**: We obtained KOOS-12 item parameter estimates from the previous linking study and existing data collected separately for the Arthritis for Shared Knowledge Study. At 12 months post-orthopaedic consult, 700 people completed both the KOOS-12 and PROMIS measures. Participants were on average 68 years old (SD = 9.3), primarily female (N = 430, 62%), and the majority chose non-surgical treatment (N = 447, 64%). Using the previously estimated KOOS item parameters we generated expected PROMIS PF scores and calculated the bias and linked score variability (SD of differences and root mean square difference [RMSD]). We also conducted a resampling study with various sample sizes to evaluate at which point linked scores exhibit stable properties.

**Results**: The KOOS-12-Function and PF scores had weaker associations (|r|= 0.73) than in the original linkage. Overall, linked scores from the KOOS-12-Function were higher than actual PROMIS scores (Δ < 1.8 T-score points) but variable (RMSD = 6.8). This was partially due to a ceiling effect for 93 people (13%) on the KOOS-12-Function. Across resampling, the median linking bias was consistent for both pattern-based and sum score crosswalks even with small group sizes, but the variability in score differences across resamples was reduced with larger samples. Notably, bias was smaller for individuals who chose non-surgical treatment compared to those who chose surgery (1.1 vs. 3.0 T-score points).

**Conclusions**: The KOOS-12-Function to PROMIS PF crosswalk exhibited acceptable psychometric properties in this new sample. It worked best with non-surgical patients, who generally exhibited better functioning. Bias was moderate on average but consistent across group sizes. Within-resample score variability was relatively wide such that score differences in any one resample required moderate group sizes (> = 30 individuals) for more stable estimates.

## O66

### Real-time symptom monitoring using ePROs to prevent adverse events during care transitions

#### Jorge Rodriguez^1^, Kaitlyn Konieczny^1^, Savanna Plombon^1^, Pamela Garabedian^1^, Maria Edelen^1^, Stuart Lipsitz^1^, Robert Rudin^2^, Anuj Dalal^1^

##### ^1^Brigham and Women's Hospital, Boston, USA. ^2^RAND Corporation, Boston, USA

*Journal of Patient-Reported Outcomes* 2023, **7(1)**: O66

**Objective**: Adverse events (AE) are common during care transitions (19–28%) in patients with multiple chronic conditions (MCC). While early indicators of post-discharge AEs include new and worsening symptoms, monitoring of patient-reported symptoms is lacking. The 21st Century Cures Act mandates the healthcare industry to adopt standardized data definitions and application programming interfaces (APIs) to support patient self-management. Health apps can individualize risk assessment and escalation of potential AEs during transitions by combining electronic health record (EHR) data with responses from patient-reported outcome (PRO) questionnaires. Communicating such information in real-time to patients, their care partners and care team may lead to earlier intervention for patients.

**Methods**: As part of our AHRQ funded study, we are developing and validating a predictive model of post-discharge AEs using selected variables derived from the 10-item Global Health PROMIS questionnaire, a validated patient-centered discharge preparation checklist, and EHR data using a structured chart review process; and designing a patient-portal integrated app using principles of user-centered design, scalability, and “techequity” to facilitate real-time symptom monitoring after discharge using electronic PROs.

**Results**: We have iterated upon preliminary requirements from 35 chart reviews and 10 structured interviews of patients and clinicians. Our proposed intervention leverages APIs to combine data collected from patient questionnaires with data retrieved from any interoperable EHR to operationalize key variables in our predictive model individualized to patients. It uses web-based modular architecture to ensure seamless integration with vendor EHRs and patient portals, and multimodal communication methods (texting, email, video) to mitigate digital divides and escalate worrisome trends to the transitional care team.

**Conclusions**: The use of electronic PROs in our patient portal-integrated app for post-discharge symptom monitoring, AE surveillance, and escalation is novel and potentially transformative–it will empower patients to understand and trend their individual risk of AEs, provide tailored self-care guidance, and help them determine when to seek help. Our approach for monitoring and escalating patient-reported symptoms for MCC patients at risk for AEs during transitions has potential to be useful for any institution with an interoperable EHR.

## P72

### Two-step screening for anxiety symptoms in kidney transplant recipients

#### Aghna Wasim^1^, Rashi Gupta^1^, Janet Li^1^, Nathaniel Edwards^1^, Madeline Li^2^, Doris Howell^3^, Susan Bartlett^4^, John Peipert^5^, Marta Novak^6^, Istvan Mucsi^1^

##### ^1^Multi-Organ Transplant Program and Division of Nephrology, University Health Network, Toronto, Canada. ^2^Department of Supportive Care, Princess Margaret Cancer Centre, Toronto, Canada. ^3^Princess Margaret Cancer Centre, Faculty of Nursing, University of Toronto, Toronto, Canada. ^4^Center for Health Outcomes Research, McGill University, Montreal, Canada. ^5^Department of Medical Social Sciences, Northwestern University, Chicago, USA. ^6^Centre for Mental Health, University Health Network, Toronto, Canada

*Journal of Patient-Reported Outcomes* 2023, **7(1)**: P72

**Objective**: Systematic screening for anxiety helps identify patients, who may benefit from clinical assessment and psychosocial support. We assess a two-step screening among kidney transplant recipients (KTR). In a pre-screening step, we compare two ultra-brief pre-screening tools (Patient-Reported Outcomes Measurement Information System Anxiety Computer Adaptive Test [PROMIS-A CAT] Screener Item: “in the past 7 days I felt anxious” versus Generalized Anxiety Disorder-2 [GAD-2]). Participants who “pre-screen positive” are assessed using PROMIS-A CAT.

**Methods**: Secondary analysis of data collected in a single center cross-sectional study in Toronto, Canada. KTRs completed the GAD-7 and PROMIS-A CAT. We also collected sociodemographic and clinical characteristics. A 2-step scenario was simulated, whereby only patients obtaining a score above a pre-screening cut-off on either of the ultra-brief pre-screeners would proceed to step 2 and complete the full PROMIS-A CAT. A score of  ≥ 10 on the GAD-7 was used to define moderate or severe anxiety. Screening performance was assessed using sensitivity and specificity.

**Results**: Of the 185 participants [mean (SD) age = 54(13) years] 57% were male and 60% were White Canadian. Based on the GAD-7, 12% had moderate or severe anxiety. Pre-screening with PROMIS-A CAT screener > “never” combined with PROMIS-A CAT ≥ 53 provided a sensitivity of 0.87 and a specificity of 0.60. Combination of PROMIS-A CAT screener item > “never” and PROMIS-A CAT ≥ 55 had the same sensitivity (0.87) and higher specificity (0.67). Pre-screening with GAD-2 ≥ 1 followed by PROMIS-A CAT ≥ 55 provided a sensitivity of 0.87 and specificity of 0.81. Compared to PROMIS-A CAT alone, the 2-step screening reduced the average number of questions patients had to complete by 44% and 30% for the PROMIS-A CAT pre-screeners and GAD-2, respectively. This reduction was most pronounced for patients with no or low anxiety.

**Conclusions**: A 2-step screening method using PROMIS-A CAT pre-screener or GAD-2 pre-screener followed by PROMIS-A CAT had good sensitivity and specificity and can help reduce question burden, particularly for patients with no or low anxiety. Clinical assessment will be required for screened-in patients to establish diagnosis of anxiety and decide on appropriate psychosocial support.

## P73

### Reha Toolbox: linking key rehabilitation measures to the PROMIS metric

#### Alexander Obbarius^1,2^, Claudia Hartmann^1^, Felix Fischer^1^, Matthias Rose^1,3^

##### ^1^Department of Psychosomatic Medicine, Center for Internal Medicine and Dermatology, Charité—Universitätsmedizin Berlin, Berlin, Germany. ^2^Dornsife Center for Self-Report Science, University of Southern California, Los Angeles, USA. ^3^Quantitative Health Sciences, Outcomes Measurement Science, University of Massachusetts Medical School, Worcester, USA

*Journal of Patient-Reported Outcomes* 2023, **7(1)**: P73

**Objective**: Researchers and clinicians assessing the health status of patients treated in rehabilitation facilities use different outcome measures. Translating scores from one measure into another would be useful for both clinical and research purposes. The aim of the Reha Toolbox study was to link three key rehabilitation measures including the World Health Organization Disability Assessment Schedule (WHODAS 2.0), the Indicators of Rehabilitation Status (IRES-3), and Hamburg Modules for the Assessment of Psychosocial Health (HEALTH-49) to the patient reported outcomes measurement information system (PROMIS) metric using item response theory (IRT).

**Methods**: Five clinicians and PRO experts mapped each item from the three rehabilitation measures to the PROMIS scales Global Health, Pain Interference, Physical Function, Dyspnea, Fatigue, Depression, Anxiety, Cognition, Ability to Participate in Social Roles and Activities, and Satisfaction with Participation in Social Roles. In a single-group design, all selected items of the rehabilitation measures were collected together with the corresponding 4-item PROMIS short forms in an online sample of N = 1000 from the general population. We tested the IRT linking assumption of construct similarity between measures by comparing item content and testing unidimensionality of items using exploratory bifactor analysis in a structural equation modeling framework. We linked rehabilitation items measuring the same construct to the PROMIS metric (item parameters fixed) using graded-response IRT models. We produced crosswalk tables for each construct by estimating expected a posteriori (EAP) scores for each sum score obtained with the rehabilitation measures. We compared the measurement precision of the rehabilitation measures and the PROMIS short-forms across the T-Score continuum.

**Results**: The number of rehabilitation items mapped to the PROMIS scales ranged between 4 (Dyspnea) and 24 (Physical Function). All constructs had sufficient unidimensionality, and all included items were successfully calibrated on the PROMIS metric. Measurement precision of the rehabilitation items across the T-Score continuum differed between constructs and number of calibrated items. Most of the PROMIS scores obtained with rehabilitation measures had a standard error of measurement of less than 0.3 across the measurement range.

**Conclusions**: We were able to generate robust linking between item subsets of WHODAS 2.0, IRES-3, HEALTH-49, and various PROMIS scales.

## O75

### Examining differential item function on PROMIS-29 between the general population and survivors of burn injury

#### Alyssa Bamer^1^, Kara McMullen^1^, Andrew Humbert^1^, Colleen Ryan^2,3^, Jeffrey Schneider^4^, Barclay Stewart^5^, Oscar Suman^6^, Nicole Gibran^1^, Kimberly Roaten^7^, Dagmar Amtmann^1^

##### ^1^University of Washington, Seattle, USA. ^2^Shriners Hospitals for Children, Boston, USA. ^3^Massachusetts General Hospital, Boston, USA. ^4^Spaulding Rehabilitation Hospital, Boston, USA. ^5^Harborview Injury and Prevention Center, Seattle, USA. ^6^University of Texas Medical Branch, Galveston, USA. ^7^University of Texas Southwestern Medical Center, Dallas, USA

*Journal of Patient-Reported Outcomes* 2023, **7(1)**: O75

**Objective**: The NIH-funded Patient-Reported Outcomes Measurement System (PROMIS) profile has been validated for use in diverse populations, including burn survivors, but Differential Item Functioning (DIF) due to burn injury has not been examined. The purpose of this study was to extend the validation of PROMIS-29 in burn survivors by examining DIF of five domains in the PROMIS-29 profile to compare burn survivors to a general population sample.

**Methods**: The PROMIS-29 domains of physical function, anxiety, depression, fatigue, and pain interference were evaluated for DIF between burn survivors and the general US population. Data from burn injury survivors was collected as part of the Burn Model System National Longitudinal Database study. Participants completed standard versions of the PROMIS-29 domains included in this study between 6 months and 20 years post-burn. The PROMIS Wave 1 publicly available dataset was used as the comparison general population sample for DIF analyses. Wave 1 participants with complete data on the four short form items within the domain were used for analyses of that domain. The software package lordif in R was used to evaluate DIF, with a pseudo R2 change of 0.02 used as criterion for identifying statistically significant DIF.

**Results**: 876 burn survivors completed at least one domain of the PROMIS-29, with the majority (n = 840) completing all domains. The majority of burn respondents were men (68%), White (69%), with mean age of 44.6 years and time since burn of 3.4 years. The number of PROMIS Wave 1 participants included in analyses was 4,052, though sample sizes for individual domain analyses ranged from 748 (fatigue) to 851 (physical function). The average age of the PROMIS sample was 51.2 years, and the majority were White (79%) and female (52%). Using the R2 criterion no items on any of the five domains were flagged for DIF.

**Conclusions**: This study provides evidence that PROMIS-29 functions the same way in burn survivors as in the general population. In combination with previous studies, these results provide support for the validity of the PROMIS-29 profile in individuals with moderate to severe burn injury for group and individual analyses.

## P76

### Psychometric analysis of PROMIS parent-proxy upper extremity short form for typically developed children aged 5–7years

#### Brittany Garcia^1,2^, Mary Claire Manske^2^, Jenny Dorich^4^, Michelle James^2^

##### ^1^University of Utah Hospital Department of Orthopaedic Surgery, Salt Lake City, USA. ^2^Shriner's Hospital for Children Northern California, Sacramento, USA. ^4^Cincinnati Children's Hospital, Cincinnati, USA

*Journal of Patient-Reported Outcomes* 2023, **7(1)**: P76

**Objective**: The Patient-Reported Outcomes Measurement Information System, PROMIS, is one widely used health status survey to evaluate health domains and has become a frequently utilized way to assess pediatric upper extremity (UE) functional outcomes.1, 2, 3, 4 The purpose of this study was to evaluate how healthy, typically developed 5–7-year-old patients perform on PROMIS parent-proxy UE short form (SF) surveys and we hypothesized they would demonstrate impairment.

**Methods**: This was a multi-center, prospective study including five children’s hospitals throughout the United States. Parents of healthy, typically developing pediatric patients aged 5–7-years-old, with no known UE diagnosis were recruited for inclusion. After compiling the 8 question PROMIS UE physical function (PF) items, mean T-scores with 95% confidence intervals were calculated for each one-year age category (5, 6, & 7 years). Univariate linear regression was used to evaluate the association between age categories and T-scores. Percentage of cohort impairment was tabulated. Ceiling and floor effects were calculated by identifying the proportion with the highest and lowest possible score.

**Results**: Parents of 162 typically developed 5–7-year-old patients completed PROMIS UE SF items. Of these, 70 (43.2%) were aged 5–5.9-years, 49 (30.2%) were 6–6.9-years, and 43 (26.5%) were 7–7.9-years-old. Of the patients 52% were female and a majority were Caucasian (65%). The mean T-score for all patients was 35.7 (± 6.7), indicating 5–7-year-old patients as a group scored in the moderate impairment region. Mean UE SF T-scores were statistically different between 5-year-old patients and the 6- and 7-year-old age categories, however 6-year-old patient’s T-scores were not statistically different than 7-year-old patients. More 5-year-old patients demonstrated moderate (27.8%) and severe (10.5%) impairment compared to older cohorts. Minimal ceiling and floor effects were demonstrated in 5- and 6-year-old patients (< 5%), while 7-year-old patients demonstrated the greatest ceiling effect (9.3%).

**Conclusions**: Typically developing 5–7-year-old patients demonstrate moderate impairment on current PROMIS Parent-Proxy UE short forms. Five-year-old patients demonstrate significantly more impairment on upper extremity short forms, than 6- and 7-year-old patients. These findings should be taken into consideration when interpreting pediatric patient UE PROMIS scores in the setting of upper extremity conditions.

## O77

### Validation of the PROMIS® Medication Adherence Scale among kidney transplant recipients on tacrolimus

#### John Peipert^1^, Courtney Hurt^2^, Richard Slay^3^, Briggs Smith^3^, George Greene^2^, John Friedewald^2^, David Cella^2^, Alison Keys^3^, David Taber^3^

##### ^1^Northwestern University, Chicago, USA. ^2^Northwestern University, Chicago, USA. ^3^Medical University of South Carolina, Charleston, USA

*Journal of Patient-Reported Outcomes* 2023, **7(1)**: O77

**Objective**: Kidney transplant recipients (KTR) take daily immunosuppression medications that almost inevitably include tacrolimus (TAC). Non-adherence to TAC is a strong predictor of graft loss. Feasible tools are needed to screen patients for TAC non-adherence. The Patient-Reported Outcome Information System (PROMIS®) Medication Adherence Scale (PMAS) was recently developed with input from KTRs. Here, we report on a psychometric evaluation of the PMAS among KTRs taking oral TAC.

**Methods**: In this prospective observational longitudinal analysis, 230 KTRs were surveyed at 2 transplant centers using the 9-item PMAS instrument assessing medication adherence (e.g., remembering to take medications, taking even when there are side effects). We compared several confirmatory factor analysis (CFA) models to examine the PMAS’s dimensionality. We created scales by summing item responses (each item has 5 response options). We calculated Cronbach’s alpha reliability of scores. Using data from one of the transplant centers, we estimated correlations between the PMAS score and a biomarker for TAC non-adherence (coefficient of variation (CV) % for TAC in the blood trough). Finally, we compared mean PMAS scale scores between patients reporting high vs. low side effect bother on Functional Assessment of Cancer Therapy item GP5 (“I am bothered by side effects of treatment”).

**Results**: We selected a 2 correlated factor CFA model (comparative fit index = 0.995; root mean squared error of approximation = 0.071; factor correlation = 0.482) that yielded two scales: Medication Beliefs & Knowledge (MBK; 4 items; range 4–20; coefficient alpha = 0.96); Medication Taking Behaviors (MTB; 5 items; range 5–25; coefficient alpha = 0.87). Higher scores indicate better medication adherence. Mean scores were: MBK = 18.8 (SD = 3.1); MTB = 24.6 (SD = 1.2). The correlation between CV% with MTB was strong at − 0.42 but was lower with MBK at − 0.26. Mean MTB scores differed significantly between patients reporting high vs. low side effect bother (23.9 vs. 24.7; *p* = 0.02; d = − 0.67).

**Conclusions**: The PMAS instrument showed preliminary reliability and validity among KTRs on oral TAC. This evidence instills confidence around the use of PMAS to screen for non-adherence in clinical settings, and as a potential outcome in studies testing adherence-promoting interventions.

## O78

### Higher PROMIS anxiety at onset of living kidney donor evaluation predicts actual donation

#### Amy Waterman^1^, Francis Weng^2^, Edward Graviss^1^, Duc Nguyen^1^, John Peipert^3^

##### ^1^Houston Methodist Hospital, Department of Surgery and J.C. Walter Jr. Transplant Center, Houston, USA. ^2^Cooperman Barnabas Medical Center, Renal and Pancreas Transplant Division or Transplant Division, Livingston, USA. ^3^Northwestern University, Chicago, USA

*Journal of Patient-Reported Outcomes* 2023, **7(1)**: O78

**Objective**: Living donor kidney transplantation (LDKT) is the optimal treatment with the best clinical outcomes for kidney failure. However, LDKT rates in the United States (US) have plateaued, and racial disparities in access to living donation (LD) have increased. Individuals considering LD may experience emotional distress around the potential for surgery or the stressors caused by undergoing evaluation and drop out. Improved understanding of ways to support potential living donors could improve access to LD for thousands. We examined which characteristics of potential living donors were associated with higher anxiety and whether higher anxiety was associated with actual LD using data from a longitudinal cohort study.

**Methods**: Potential living donors from 5 transplant centers in the US were surveyed prior to beginning their medical evaluation. Survey measures included assessments of potential donors’ attitudes, motivations, concerns, and knowledge around the donation process, including a custom short form containing 4 items from PROMIS Item Bank v1.0—Anxiety. Then, participants were followed through evaluation for up to 12 months to determine if they ultimately donated a kidney or not. We used multivariable logistic regression models to identify the characteristics associated with higher anxiety (T score > 55) and kidney donation.

**Results**: In total, 2184 individuals were surveyed, of which 407 (18.6%) ultimately donated their kidney. The median PROMIS Anxiety T score for the entire sample was 46.8 (IQR: 39.4, 54.8); 19.7% (n = 424) of these had T scores > 55. Having someone important who did not support the donation [odds ratio (OR): 2.25; 95% CI: 1.29, 3.95] and having an intended recipient who is a close family member (OR: 1.72; 1.07, 2.79) were associated with higher anxiety. Anxiety T scores did not vary by race/ethnicity (*p* = 0.77). An Anxiety T score of > 55 was associated with 39% reduction in odds of kidney donation (OR: 0.61; 95% CI: 0.38, 0.97).

**Conclusions**: Higher anxiety, particularly when the kidney patient is a family member, is associated with a reduced likelihood of donating. PROMIS Anxiety should be considered as a screening tool for potential living donors. Interventions to reduce anxiety among potential living donors may increase the chances of ultimately donating.

## P79

### Evaluating PROMIS preference scoring system (PROPr) in patients with liver transplant

#### Jing Zhang^1^, Maria Pucci^1^, Evan Tang^1^, Nazia Selzner^1^, Janel Hanmer^2^, Barry Dewitt^3^, John D. Peipert^4,5^, Ron D. Hays^6^, Istvan Mucsi^1^

##### ^1^Ajmera Transplant Centre, University Health Network, Toronto, Canada. ^2^Department of General Internal Medicine, University of Pittsburgh Medical Center, Pittsburgh, USA. ^3^Department of Engineering & Public Policy, Carnegie Mellon University, Pittsburgh, USA. ^4^Department of Medical Social Sciences, Northwestern University, Chicago, USA. ^5^Northwestern University Transplant Outcome Research Collaborative, Chicago, USA. ^6^Department of Medicine, UCLA, Los Angeles, USA

*Journal of Patient-Reported Outcomes* 2023, **7(1)**: P79

**Objective**: PROPr is a preference-based health state summary score within the Patient Reported Outcome Measurement Information System (PROMIS®). We aimed to assess the construct validity of PROPr among liver transplant recipients (LTR) using the “legacy” instruments EQ-5D-5L and Short-Form Six Dimension (SF-6D®).

**Methods**: A cross-sectional, single-center sample of adult LTRs completed questionnaires including PROMIS-29 or PROMIS-29 + 2 (v 2.1) or PROMIS-CAT, EQ-5D-5L, the Liver Disease Quality of Life questionnaire (LDQOL), Patient Health Questionnaire-9 (PHQ-9) and Edmonton Symptom Assessment Scale-revised (ESASr). SF-6D was calculated from the SF-12, PROPr was generated from PROMIS domain scores, EQ-5D-5L utility score was calculated using the Canadian value set. Convergent validity was assessed using Pearson’s correlation between PROPr vs EQ-5D-5L and SF-6D. Construct validity was evaluated using “clinical condition impacts”, that is the coefficient for a health condition when summary score was regressed on the health condition in a univariable regression analysis. PROPr and legacy measures were compared between groups formed by clinical variables (comorbidity, symptom burden, low serum albumin, anemia, diabetes), that were expected to have different impacts on health-related quality of life.

**Results**: Mean ([Standard deviation] SD) age of the 200 participants was 56 (15) years, 67% were male and 73% Caucasian. PROPr and SF6D scores were less subject to ceiling effects than the EQ-5D-5L. Strong correlations were observed between PROPr and EQ-5D-5L (r = 0.68) and SF-6D (r = 0.79). PROPr demonstrated a larger impact estimate for all known health conditions compared to both the SF-6D and the EQ-5D-5L. Condition impact for PROPr was: moderate/severe versus no/mild depressive symptoms (− 0.33, *P* < 0.001), none/mild vs moderate/severe symptom burden (− 0.32, *P* < 0.001) and anemia vs normal hemoglobin level (− 0.08, *P* = 0.050).

**Conclusions**: These results support the validity of PROPr use among patients with liver transplantation. Moreover, PROPr may be more sensitive to differences in health states than the EQ-5D-5L and the SF-6D.

## P80

### Assessing pain among solid organ transplant recipients using PROMIS tools

#### Alyssa Yantsis^1,2^, Liza Markova^1^, Jenny Kang^1^, Pearse O'Malley^1^, Madeline Li^3^, Doris Howell^4^, Susan Bartlett^5^, John Peipert^6^, Marta Novak^7^, Istvan Mucsi^1^

##### ^1^Ajmera Transplant Program and Division of Nephrology, University Health Network, Toronto, Canada. ^2^University of Toronto, Temerty Faculty of Medicine, Toronto, Canada. ^3^Department of Supportive Care, Princess Margaret Cancer Centre, Toronto, Canada. ^4^Princess Margaret Cancer Centre, Faculty of Nursing, University of Toronto, Toronto, Canada. ^5^Center for Health Outcomes Research, McGill University, Montreal, Canada. ^6^Department of Medical Social Sciences, Northwestern University, Chicago, USA. ^7^Centre for Mental Health, University Health Network, Toronto, Canada

*Journal of Patient-Reported Outcomes* 2023, **7(1)**: P80

**Objective**: Pain is often associated with poorer quality-of-life and health amongst solid organ transplant (SOT) recipients. In addition, a complex, bidirectional relationship exists between pain and depression among chronically ill patients. We assess the association between pain intensity (PI) and pain interference (PIF) while controlling for co-variables, including depressive symptoms, among SOT recipients.

**Methods**: Secondary analysis of a single-centre, cross-sectional convenience sample of adult SOT recipients. Participants completed the Patient-Reported Outcome Measurement Information System (PROMIS)-29 item profile or PROMIS computer adaptive tests (CATs) with the same domains. Pain was assessed using the single 0–10 numeric item (PI) and PROMIS Pain Interference (PIF) T-score. PI was categorized as: No = 0; Mild = 1–3; Moderate/Severe = 4–10. Multivariable-adjusted linear regression was performed to assess associations between PI, clinical, socio-demographic variables, depression and PIF. We also assessed associations between these variables and moderate/severe PIF (T-score > 60).

**Results**: Of 581 participants, 381 were kidney (KT), 47 kidney-pancreas (KP), and 153 liver (LT) transplant recipients. Mean(SD) age was 52(15), 62% were male, and 63% were white. Median(IQR) of years since transplant was 8(13) for KT, 7(10) for KP, and 5(11) for LT. LT had a higher median(IQR) PI score compared to KT (2[4] vs.1[3], *p* < 0.001) and had a greater proportion of moderate/severe PI compared to KPs and KTs (31% vs. 28% vs.19%, *p* < 0.001). PIF was not different between SOT types. PI correlated strongly with PIF scores (r = 0.745, *p* < 0.001). The association between PI and PIF scores remained significant (Coeff = 2.818, *p* < 0.001; 95% CI: 2.574–3.062) in multivariable-adjusted linear regression model. Median(IQR) PIF was higher for moderate/severe PI compared to mild and no PI among SOT recipients (62[9] vs. 53[9] vs. 39[2], *p* < 0.001). In a multivariable adjusted logistic regression model, moderate/severe PI was associated with greater odds of moderate/severe PIF (OR:20.899, *p* < 0.001; 95% CI: 11.656–37.472). Both PI (r = 0.304) and PIF (r = 0.392) were correlated with depression (*p* < 0.001). Depression remained significantly associated with PI and PIF in multivariable linear and logistic models.

**Conclusions**: The relationship between PI and PIF is complex. LT recipients report more severe pain compared to others. Future analyses will further explore pain and depression.

## P81

### Association of PROMIS physical function and health-related quality of life among solid organ transplant recipients

#### Wajiha Ghazi^1,2^, Cindy Wen^1^, Hiba Alhabbal^2^, Princess Okoh^1,2^, John Peipert^3^, Susan Bartlett^4^, Madeline Li^5^, Doris Howell^6^, Marta Novak^7^, Istvan Mucsi^1,2^

##### ^1^Ajmera Transplant Program, University Health Network, Toronto, Canada. ^2^University of Toronto, Toronto, Canada. ^3^Feinberg School of Medicine, Northwestern University, Chicago, USA. ^4^Center for Health Outcomes Research, McGill University, Montreal, Canada. ^5^Department of Supportive Care, Princess Margaret Cancer Centre, Toronto, Canada. ^6^Princess Margaret Cancer Centre, Faculty of Nursing, Toronto, Canada. ^7^Centre for Mental Health, University Health Network, Toronto, Canada

*Journal of Patient-Reported Outcomes* 2023, **7(1)**: P81

**Objective**: Ability to perform activities of daily living is a critical outcome for solid organ transplant (SOT) recipients. Many of these patients have low physical function (PF) which is associated with poor quality of life and clinical outcomes. We assess the association of PF with health-related quality of life among SOT recipients.

**Methods**: A secondary analysis was conducted with data obtained from a cross-sectional convenience sample of SOT (kidney, kidney-pancreas and liver) recipients. Participants completed PROMIS PF item bank (4 item short form or Computer Adaptive Tests), EQ-5D-5L and sociodemographic questionnaires. PROMIS PF T-scores were categorized: ‘no/mild’ (> = 45), ‘moderate’ (40–45), and ‘severe’ (< 40) impairment. We recoded the responses to the EQ-5D-5L mobility domain as ‘no problem’ versus ‘any problems.’ Independent associations between EQ5D utility score (outcome) and the PROMIS PF T-score and physical impairment groups (exposures) were tested in linear regression models adjusted for sociodemographic and clinical variables. Discrimination of PROMIS PF was assessed using the Receiver Operating Characteristic (ROC).

**Results**: Of 692 participants, mean (SD) age was 52(15) years with 63% male, median (Interquartile Range(IQR)) years since transplant was 6.5 (12.1). 53% of the sample had no/mild impairment, 20% had moderate, and 27% were severely impaired in PF. Median(IQR) PF T-scores were higher for patients who reported ‘no problems’ vs those who indicated moderate or severe mobility problems on the EQ-5D-5L domain: [50(11) vs 40(7) vs 33(6)]. PROMIS PF showed excellent discrimination for impaired mobility (ROC = 0.86,95% CI: 0.83–0.89). Median(IQR) EQ5D utility scores were higher for patients with PF T-scores >  = 45 vs those with scores 40–45 and < 40 [0.91(8) vs 0.85(0.1) vs 0.73(0.28)]. In multivariable adjusted linear regression, higher EQ5D utility scores were associated with higher PROMIS PF scores (B = 0.01, *p* < 0.001;95% CI: 0.009–0.012). Similarly, lower EQ5D utility scores were associated with moderate (B = − 0.07, *p* < 0.001; 95% CI: − 0.1 to − 0.04) and severe PF impairment (B = − 0.22, *p* < 0.001; 95% CI: − 0.24 to − 0.19).

**Conclusions**: PROMIS PF was associated with health-related quality of life in SOT recipients. In future research, we will explore additional ways to improve interpretability of PROMIS PF scores in transplant recipients.

## O82

### Two-step screening for depressive symptoms in solid organ transplant recipients

#### Tibyan Ahmed^1^, Nathaniel Edwards^1^, Fazeela Amiri^1^, Cathy Yang^1^, Heather Ford^1^, John D. Peipert^2^, Susan J. Bartlett^3^, Madeline Li^4^, Doris Howell^5^, Marta Novak^6^, Istvan Mucsi^1^

##### ^1^Ajmera Transplant Centre, University Health Network and University of Toronto, Toronto, Canada. ^2^Feinberg School of Medicine, Northwestern University, Chicago, USA. ^3^Centre for Health Outcomes Research, McGill University, Montreal, Canada. ^4^Department of Supportive Care, Princess Margaret Cancer Centre, Toronto, Canada. ^5^Lawrence S. Bloomberg Faculty of Nursing, University of Toronto, Toronto, Canada. ^6^Centre for Mental Health, University Health Network, Toronto, Canada

*Journal of Patient-Reported Outcomes* 2023, **7(1)**: O82

**Objective**: Patients with end-stage organ failure who undergo solid organ transplant (SOT) often experience depressive symptoms. However, these symptoms are frequently undetected. Systematic screening for depressive symptoms may identify patients who may benefit from additional psychosocial support and clinical assessment. Here we assess a two-step method using (1) ultra-brief pre-screeners (Patient- Reported Outcomes Measurement Information System Depression Computer Adaptive Test Screener Item “in the past 7 days I felt depressed” [PROMIS-D CAT_s_] or Patient-Health-Questionnaire-2 [PHQ-2]; followed by (2) PROMIS-D CAT, to screen for depressive symptoms in SOT recipients.

**Methods**: We performed secondary analysis of data collected from a single center cross-sectional study in Toronto, Canada. We simulated 2-step screening scenarios where only patients above a pre-screening cut-off score would subsequently complete step 2 (PROMIS-D CAT in its entirety). Screening performance was evaluated by sensitivity, specificity, positive and negative predictive values (PPV and NPV). A Patient Health Questionnaire-9 (PHQ-9) score of ≥ 10 was used as the referent to identify patients with moderate/severe depressive symptoms. Sociodemographic and clinical characteristics were also collected.

**Results**: Of 285 participants, the mean (SD) age was 52 (15), 66% were male and 67% were White Canadian. Based on PHQ-9, 18% of patients had moderate/severe depressive symptoms. Pre-screening with PROMIS-D CAT_s_ > “never” combined with PROMIS-D CAT ≥ 53 provided good sensitivity (sensitivity: 0.80, specificity: 0.76, PPV: 0.42, NPV: 0.95). Using PHQ-2 ≥ 1 followed by PROMIS-D CAT ≥ 53 had somewhat higher specificity (sensitivity: 0.78, specificity: 0.81, PPV: 0.48, NPV: 0.95). Compared to administering PROMIS-D CAT alone, the 2-step method reduced the average number of questions patients had to complete by 53% and 31% for PROMIS-D CAT_s_ and PHQ-2, respectively. This reduction was most pronounced among patients with no or low level of depressive symptoms.

**Conclusions**: A 2-step screening method using PROMIS-D CAT_s_ pre-screener followed by PROMIS-D CAT in its entirety had good sensitivity and moderate specificity, and can be most helpful to reduce question burden among patients with no depressive symptoms. Screened in patients will require further clinical assessment to establish diagnosis and decide on appropriate psychosocial support.

## O84

### The PROMIS Pediatric Item Banks Norming Project

#### Jin-Shei Lai, Xiaodan Tang, Benjamin D. Schalet, Michael A. Kallen, Aaron J. Kaat, David Cella

##### Northwestern University, Chicago, USA

*Journal of Patient-Reported Outcomes* 2023, **7(1)**: O84

**Objective**: The PROMIS Pediatric Item Banks (Anger, Anxiety, Depressive Symptoms [DS], Fatigue, Peer Relationships [PR], Mobility, and Upper Extremity Function [UE]) were developed more than one decade ago using data from clinical samples and the US general population. Here we report updated item parameters and reference scores for these item banks.

**Methods**: Participants included 1,016 children (ages 8–17 years) drawn from a probability-based US general population panel collected in 2021–2022. Participants were randomly assigned to one of two forms and completed full-length item banks allocated to either form. Unidimensionality was evaluated using confirmatory factor analysis or bi-factor analysis. Criteria were: (a) Comparative Fit Index > 0.9, (b) Root Mean Square Error of Approximation < 0.1, (c) R2 > 0.4, (d) residual correlations < 0.2, and for bi-factor models only, (e) factor loadings on specific factors < general factor, and (f) variance explained by the general factor > specific factors. Differential item functioning (DIF) was evaluated due to age, gender, race, and household income. Parameters of items that fit the IRT model (i.e., χ^2^/df < 3.0) were estimated using the graded response model. For Mobility and UE, multi-group calibration analyses were employed; specifically, both data from the current sample and the original PROMIS Wave 1 sample were used and final parameters were centered on the current norming sample. Finally, rescaling approaches were used so that norms of the general population were 50 (in T-score) on all measures.

**Results**: Several items were removed prior to calibration (content appropriateness: 1 in PR, 3 in Mobility, 2 in UE; non-unidimensional: 2 in UE; DIF: 1 in UE). All remaining items fit the IRT model. IRT-scaled scores using the newly estimated parameters had higher theta values than those using current parameters with the discrepancies (new—current) ranging from 0.34 to 1.15 (in theta).

**Conclusions**: Using new data from the US general population, we have refined pediatric item banks, re-estimated item parameters, and established new reference values centered around T score = 50. Parallel efforts were made on corresponding proxy versions. Both child- and proxy-reports will be available in Healthmeasure.net in Fall 2022.

## P85

### Higher levels of epilepsy related stigma were found amongst foreign-born adults with epilepsy in Sweden

#### Klara Andersson^1,2,3^, Anneli Ozanne^1,3^, Johan Zelano^2,3^, Kristina Malmgren^2,3^, John E. Chaplin^4^

##### ^1^Institute of Health and Care Sciences, Sahlgrenska Academy, University of Gothenburg, Gothenburg, Sweden. ^2^Institute of Neuroscience and Physiology, Sahlgrenska Academy, University of Gothenburg, Gothenburg, Sweden. ^3^Department of Neurology, Sahlgrenska University Hospital, Gothenburg, Sweden. ^4^Department of Pediatrics, Institute of Clinical Sciences, University of Gothenburg, Gothenburg, Sweden

*Journal of Patient-Reported Outcomes* 2023, **7(1)**: P85

**Objective**: Stigma significantly contributes to the burden of disease in epilepsy. This study investigated the association between stigma, country of birth and mental health.

**Methods**: In this observational study adults with a diagnose of epilepsy (defined as having an ICD-10 code of G40 in the patient journal) and no cognitive impairment were included from three neurology out-patient clinics in the southwest of Sweden with different patient catchment profiles. Patients completed the HADS anxiety and depression scales, NeuroQOL stigma short-form and the PROMIS Global Health 1.2 to assess mental health. All questionnaires were available in English, Swedish and Arabic. The scales that were not previously translated to Swedish and Arabic were translated and validated through a face-to-face validation process before the study. Questions of demographic characteristics, seizures, stigma and mental health generated categorical and continuous variables that were analyzed with Independent samples T-test, ANOVA, Pearson correlation, Fisher’s exact test and a stepwise multiple regression using SPSS version 28.

**Results**: In total 161 adults with epilepsy were included in the cohort. The mean NeuroQOL stigma score was 48.3(sd 8.1) and the mean PROMIS Mental Health was 44.5(sd 9.9). Non-European born participants reported a higher stigma score than native-born participants (52.3 compared to 47.0, *p* = 0.003). Active seizures were noted more frequently in non-European born participants, however the difference did not reach statistical significance (61.8% compared to 51.3%). In the total cohort, a higher NeuroQOL stigma score was associated with a lower PROMIS mental health score (− 0.25, *p* = 0.001). The NeuroQOL stigma score was also significantly associated with seizure frequency last year, having seizures in public, country of birth, HADS anxiety and HADS depression. Following multiple regression analysis only three variables remained significantly associated with NeuroQOL: seizure frequency, HADS anxiety and PROMIS mental health.

**Conclusions**: Stigma scores were significantly higher in the non-European-born adult epilepsy patients in Sweden. Sample size, selection bias and the complex nature of multiple sources of stigma need to be considered, however, our study underscores the importance of assessing not only seizure frequency, but also anxiety and mental health in this patient group.

## P86

### Cultural adaptation and linguistic validation of English PROMIS measures in India

#### Helena Correia, Jiyoung Son, Emna Maksud, David Cella

##### Northwestern University, Chicago, USA

*Journal of Patient-Reported Outcomes* 2023, **7(1)**: P86

**Objective**: The purpose of this study was to assess if the English version of selected PROMIS measures is appropriate for English speakers in India through linguistic validation, and to adapt the wording where necessary to produce a culturally appropriate version.

**Methods**: The following measures were cognitively debriefed in India: PROMIS SF v2.0—Physical Function 10b, PROMIS SF v2.0—Physical Function 8c, PROMIS SF v1.0—Fatigue 8a, PROMISnq SF v2.0—Physical Function-MS 15a, PROMIS Scale v1.2—Global Health, PROMIS-29 Profile v2.1, PROMIS Pediatric-25 Profile v2.0, PROMIS Parent Proxy-25 Profile v2.0, and PROMIS Early Childhood Parent Report Scale v1.0—Global Health 8a. Each measure was debriefed with five native-speaking participants from the general population, to evaluate comprehensibility and appropriateness of the items. Linguistic equivalence and wording appropriateness of each item were determined by conducting a qualitative analysis of participants’ comments.

**Results**: The measures for adults were completed and debriefed with a total of 35 adults, and the pediatric profile was tested with five children ages 9 to 16. All participants were from the general population and born in India. Cognitive interviews revealed that several concepts were not well understood by some participants and required revisions: e.g., “it was hard to” was revised to “it was difficult to”, as the word “hard” is uncommonly used to convey the meaning of difficulty; “run errands and shop” was revised to “shop and do other tasks outside the home”; “physically drained” was added to “run-down” to improve understandability. The revised wording was approved by PROMIS investigators to ensure that the adapted items retained the intended original meaning.

**Conclusions**: These nine measures are easy to understand and culturally appropriate for use in India. Most items retained their original wording. An English (India) version was created only for measures containing at least one item that required an adaptation. All English (India) measures are conceptually equivalent to the original English.

## O87

### Unidimensional versus multidimensional calibration and assessment with inter-correlated pediatric item banks

#### Michael Kallen, Jin-Shei Lai

##### Northwestern University, Chicago, USA

*Journal of Patient-Reported Outcomes* 2023, **7(1)**: O87

**Objective**: We studied unidimensional (UD) versus multidimensional (MD) calibration and assessment to learn which environment might offer enhanced measurement and/or performance characteristics when utilizing inter-correlated item banks.

**Methods**: Working in UD and MD environments with previously collected data, we calibrated three Neuro-QoL pediatric banks [Anger, Anxiety, Depression (8, 19, and 18 items, respectively)] and calculated full bank scores. We compared mean/median slope estimates and their differences across environments and determined the count/percentage of slope parameters > 4. We investigated full bank score ranges/variability and potential floor-effect case treatment (i.e., for those with extreme-low symptom status). We simulated MD- and UD-CATs with our real data and obtained (a) mean/median number of items administered, (b) CAT score ranges/variability and potential floor-effect case treatment, and (c) mean/median score SE and percentage of cases with SE < 0.4.

**Results**: Our sample contained N = 513 parent-proxy responders (n = 455 with complete item data). Mean/median MD-slope (across banks) was 2.64/2.66 versus 3.34/3.26 (UD-slope); mean UD- minus MD-slope differences were 1.13, 0.48, and 0.76 (Anger, Anxiety, Depression, respectively). N = 1 (2.2%) MD-slope was > 4 (Anger); n = 10 (22%) UD-slopes exceeded criterion (Anger-4, Anxiety-1, Depression-5). Full MD versus UD bank scores ranged from − 1.90/ + 3.91 versus − 1.25/ + 2.74 (Anger, SD = 1.18 vs. 0.91), − 2.02/ + 4.12 vs. − 1.29/ + 3.30 (Anxiety, SD = 1.22 vs. 1.00), and − 2.18/ + 4.37 vs. − 1.81/ + 3.28 (Depression, SD = 1.26 vs. 1.00). Potential floor-effect cases were better distributed with MD- than UD-scores, alleviating floor effects. Mean/median MD-CAT length (across banks) was 14.61/8.00 vs. 15.09/10.00 (UD-CAT); n = 248 (54.5%) individual MD-CATs were shorter than UD-CATs. MD-CAT scores exhibited extended scores ranges, increased variability, and better distributed potential floor-effect case treatment versus UD-CAT scores, as observed with MD full bank scores. Mean/median MD- versus UD-CAT score SE was 0.29/0.27 versus 0.37/0.35 (Anger), 0.38/0.38 versus 0.38/0.38 (Anxiety), and 0.34/0.34 versus 0.38/0.37 (Depression); percentage of MD versus UD cases with SE < 0.4 was 86.6 versus 82.4 (Anger), 81.8 versus 72.5 (Anxiety), and 89.9 versus 77.6 (Depression).

**Conclusions**: In our study, the MD calibration and assessment environment offered numerous measurement and performance-related improvements versus the UD environment. Such improvements should be confirmed for the studied Neuro-QoL pediatric banks using independent datasets and further investigated with other inter-correlated banks.

## P88

### Relationship between preoperative expectations and physical function scores at 12 months after cervical spine surgery

#### Jacquelyn S. Pennings, Jordan Bley, Rogelio A. Coronado, Kristin R. Archer

##### Vanderbilt University Medical Center, Nashville, USA

*Journal of Patient-Reported Outcomes* 2023, **7(1)**: P88

**Objective**: The objective was to examine the effects of preoperative expectations of elective cervical spine surgery on postoperative PROMIS physical function (PF) scores 12 months after surgery.

**Methods**: The 20-item HSS Cervical Spine Surgery Expectations Survey was incorporated into a spine outcomes registry (from 2018–2021) at a single center to preoperatively measure patient's expectations for pain, personal daily activities, psychosocial issues, physical function, and skeletal function for their elective spine surgery. Patients were undergoing elective cervical spine surgery for degenerative reasons. Patient demographics, clinical and surgery data, and patient reported outcomes including the PROMIS-29 are collected from medical records and patient self-report preoperatively and after surgery (3 and 12 months).

**Results**: N = 352 patients were included in the analysis. Mean age was 56.1 (SD = 11.7), 47% female, 20% undergoing a revision surgery, 65% undergoing ACDF (vs. posterior laminectomy and/or fusion). Preoperative expectations ranged from 0 to 100 (M = 67.8, SD = 23.1). PROMIS PF t-scores were M = 38.3 (SD = 6.5) at preop and M = 43.4 (SD = 9.5) at 12-months postop. Linear regression revealed that preoperative expectations were a significant positive predictor of 12-month PF t-scores (Beta = 0.08, 95%CI = 0.04 to 0.11, *p* < 0.001, Std Beta = 0.12) while controlling for preoperative PF and demographic and clinical characteristics. A non-linear relationship between preop expectations and postoperative PF revealed that for those with better physical function (t-score > 35), the positive relationship between preoperative expectation and postoperative PF was stronger and statistically significant while for those with a PF t-score of < 35 at preop, there was not a significant relationship between preoperative expectations and postoperative outcomes.

**Conclusions**: The relationship between a patient's expectations of success going into surgery and their outcomes after surgery has not been systematically evaluated using a validated expectations instrument. It is important for surgeons to understand, and perhaps even guide a patient to appropriate expectations going into elective spine surgery. Overall, patients with higher expectations before surgery tended to have better physical function 12-months after surgery. However, when examining those with very poor functioning preoperatively, we found that the relationship between expectations and postop outcomes was much weaker.

## O90

### PROMIS emotional functioning among cognitively healthy and cognitively impaired older adults

#### Manrui Zhang^1^, Emily Ho^1^, Cindy Nowinski^1^, Rina Fox^2^, Ezgi Ayturk Ergin^3^, Miriam Novack^1^, Hiroko Dodge^4^, Sandra Weintraub^1^, Richard Gershon^1^

##### ^1^Northwestern University, Chicago, USA. ^2^University of Arizona, Tempe, USA. ^3^Koc University, Istanbul, Turkey. ^4^Oregon Health & Science University, Portland, USA

*Journal of Patient-Reported Outcomes* 2023, **7(1)**: O90

**Objective**: Emotional functioning is influenced by both normal aging processes and pathological brain changes due to neurodegenerative diseases. To enable early intervention and care planning, it is important to characterize different aspects of emotional functioning among cognitively healthy older adults and older adults with early stages of cognitive impairment. Based on a sample of 448 participants aged 65 and older, we explored different aspects of emotional functioning across adults who are cognitively healthy (Normal Control (NC) = 276), and adults with Mild Cognitive Impairment (MCI = 103) or mild dementia of the Alzheimer type (mild DAT = 69). We administered PROMIS Emotion Measures, including Negative Affect and Psychological Well-being measures. Statistically significant differences were found among older adults in the NC, MCI, and mild DAT groups in Depression and Negative Affect. Among male participants, the mild DAT group showed significantly higher Anger-Physical Aggression than the NC group. The mild DAT group also showed significantly higher Anxiety and lower General Self-Efficacy than the MCI group, but only among the oldest old (above age 80). Our findings suggest the PROMIS Emotion Measures can be a useful tool for assessing emotional health among older adults with and without MCI or mild DAT.

The objective is to assess multidimensional aspects of emotional functioning in a sample of cognitive aging participants as part of a large cross-sectional, multi-cohort study using PROMIS Emotion measures.

**Methods**: A sample of 448 individuals diagnosed with either normal cognitive functioning, mild cognitive impairment (MCI) or mild dementia of the Alzheimer type (DAT) completed a set of PROMIS Emotion measures. Univariate analyses (e.g., ANOVAs) were conducted on to assess the impact of covariates such as gender and age.

**Results**: The mild DAT and MCI groups had significantly higher levels of Negative Affect and Depression compared to the NC group, while these clinical groups showed non-significant difference in Psychological Well-being. Male participants with MCI showed higher Anger than males in the NC group. Among participants older than 80, the mild DAT group had higher Anxiety and lower General Self-Efficacy than the MCI group.

**Conclusions**: These baseline differences established in this study using PROMIS Emotion measures are important for examining longitudinal trajectories of emotional health across these three clinical groups in the future.

## O91

### The responsiveness and meaningful interpretation of the PROMIS Fatigue 13a and 10a in lupus populations

#### Paul Kamudoni^1^, Alexandra Lauer^1^, Oliver Guenther^1^, Cristina Vazquez-Mateo^2^, Karon Cook^3^

##### ^1^Merck Healthcare KgaA, Darmstadt, Germany. ^2^EMD Serono Research & Development Institute, Inc., an affiliate of Merck Healthcare KgaA, Billerica, USA. ^3^Feral Scholars, Broaddus, USA.

*Journal of Patient-Reported Outcomes* 2023, **7(1)**: O91

**Objective**: Fatigue is among the most prevalent symptoms of systemic lupus erythematosus (SLE) and is associated with patient distress, work dysfunction, and worse overall health status. The National Institutes of Health (NIH) PROMIS Fatigue item bank and related short forms have advanced the measurement of fatigue across rheumatologic and other chronic conditions. The aims of this study were to evaluate the responsiveness of the PROMIS Fatigue 13a and 10a scores and to establish minimal important difference (MID) estimates in SLE populations.

**Methods**: Pooled data across treatment arms from a 52-week Phase II, placebo-controlled, randomized clinical trial evaluating evobrutinib in SLE were used in this post-hoc analysis (MS200527-0018; NCT02975336). Study participants met at least 4 of 11 American College of Rheumatology SLE criteria and had an SLE Disease Activity Index (SLEDAI-2K) score of ≥ 6. Responsiveness and MID were analyzed based on score change from baseline to week 52, using an anchor-based approach.

**Results**: At baseline, study participants (n = 466) had a mean (standard deviation, SD) age of 40 (12.3) years and 94% were female. Means (SD) scores at baseline were 55.5 (8.03), and 55.9 (7.99) for the PROMIS Fatigue 10a and 13a, respectively. Six suitable anchors were identified and used in the responsiveness analyses. The PROMIS Fatigue 13a and 10a scores were highly sensitive to both worsening and improvements in fatigue over 52 weeks (standardized response mean > 0.3 on all six anchors for worsening and on five anchors for improvement). Score changes of 2.6–4.7 (2.2–5.4) on the PROMIS Fatigue 13a and 2.5–4.4 (2.5–5.6) on the PROMIS Fatigue 10a are proposed as MID criteria for worsening (improvement) in fatigue over 52 weeks.

**Conclusions**: This research extends the evidence underpinning the applicability of the PROMIS Fatigue 13a and 10a in SLE routine clinical practice and research. The MID estimates established will aid the integration of PROMIS fatigue scores into clinical decision-making and facilitate clinician-patient communication.

